# Multiple environmental factors, but not nutrient addition, directly affect wet grassland soil microbial community structure: a mesocosm study

**DOI:** 10.1093/femsec/fiad070

**Published:** 2023-06-24

**Authors:** Keith R Edwards, Jiří Bárta, Jiří Mastný, Tomáš Picek

**Affiliations:** Department of Ecosystem Biology, Faculty of Science, University of South Bohemia in České Budějovice, Branišovská 1760, 37005 České Budějovice, Czech Republic; Department of Ecosystem Biology, Faculty of Science, University of South Bohemia in České Budějovice, Branišovská 1760, 37005 České Budějovice, Czech Republic; Department of Ecosystem Biology, Faculty of Science, University of South Bohemia in České Budějovice, Branišovská 1760, 37005 České Budějovice, Czech Republic; Department of Ecosystem Biology, Faculty of Science, University of South Bohemia in České Budějovice, Branišovská 1760, 37005 České Budějovice, Czech Republic

**Keywords:** *Carex acuta*, context dependence, r/K strategies, soil microbial community structure, wet grasslands

## Abstract

Nutrient addition may change soil microbial community structure, but soil microbes must simultaneously contend with other, interacting factors. We studied the effect of soil type (peat, mineral), water level (low, high), and nutrient addition (unfertilized, fertilized) on wet grassland soil microbial community structure in both vegetated and un-vegetated soils after five years of treatment application in a mesocosm, using Illumina sequencing of the bacterial V4 region of the small ribosomal sub-units. Soil type, water level, and plant presence significantly affected the soil microbial structure, both singly and interactively. Nutrient addition did not directly impact microbiome structure, but acted indirectly by increasing plant biomass. The abundance of possible plant growth promoting bacteria and heterotrophic bacteria indicates the importance of bacteria that promote plant growth. Based on our results, a drier and warmer future would result in nutrient-richer conditions and changes to microbial community structure and total microbial biomass and/or abundances, with wet grasslands likely switching from areas acting as C sinks to C sources.

## Teaser

Many factors, both alone and their interactions, affect soil microbial community structure and their potential functions in wetlands, most notably water level.

## Introduction

Soil microbes are an important component of the tightly connected plant-soil-microbe (PSM) system (Pugnaire et al. 2019). PSM systems in general and the soil microbial community in particular are influenced by both biotic and abiotic factors, which are known to greatly influence the abundance, community structure, and activities of soil microbes. These in turn can greatly impact plant growth and composition and, therefore, both directly and indirectly influence ecosystem functioning (Ehrenfeld et al. 2005, Chroňáková et al. 2019).

Abiotic factors, such as nutrient levels, hydrology, and soil type, are known to greatly influence soil microbe diversity and abundance (Hartman and Tringe 2019, Zhao et al. 2019). Soil nitrogen (N) contents positively affect nitrification and denitrification rates, while the abundance of ammonia-oxidizing bacteria is greater in N-fertilized conditions (Carey et al. 2016, Nguyen et al. 2018). Changes in site hydrology can impact the amount of oxygen available for aerobic metabolism (Wang et al. 2019). Flooding can lead to increased metabolism and greater abundance of the anaerobic methanogenic community (Watanabe et al. 2020), while having a positive relationship on polyphenol oxidase and peroxidase activities (Cao et al. 2020). In contrast, extensive drought can significantly negatively affect soil microbial community composition and enzymatic activities because low-water activity denatures enzymes, thereby decreasing the solubility of nutrients leading to reduced plant growth and production (Nguyen et al. 2018). Bacterial taxa may be highly correlated with certain soil characteristics, including the C:N ratio (Kuramae et al. 2011) and pH (Hansel et al. 2008). Microbial composition also can differ greatly between the rhizosphere and bulk soil, indicating the influence of the large spatial heterogeneity of soils as well as the great impact of plants on the soil microbial community (Berg and Smalla 2009, Schreiter et al. 2014).

There have been many studies on the effects of these different factors on soil microbial community structure, but most have treated these factors in isolation, as single-factor investigations (Sierra et al. 2015). However, soil microbes must contend with multiple, interacting factors, several of which may change simultaneously (Sierra et al. 2015, Reese et al. 2018). Therefore, multivariable studies may provide a more realistic outlook on how soil microbial structure and functions may be affected under changing conditions. Determining the impact of such external drivers is a necessary step in understanding how ecosystem functions may change as environmental conditions change (Smith-Ramesh and Reynolds 2017; Bennett et al. 2018; DeLong et al. 2019).

Wet grasslands are diverse ecosystems within agricultural landscapes, being transitional between littoral, permanently flooded wetlands and upland grasslands. They may be found on peat or mineral substrates, but have similar hydrologic characteristics and are maintained by disturbance (Joyce et al. 2016). Wet grasslands are open, graminoid-dominated habitats that provide many ecosystem services, including nutrient removal, flood attenuation, and groundwater recharge, as well as being important bird habitats (Joyce 2014). In Europe, wet grasslands are created and maintained by human activities, usually in the form of cutting for hay (Tallowin and Jefferson 1999). This disturbance helps to remove plant biomass, thereby reducing nutrient inputs, which allows for the co-existence of less competitive plant species. The important role of cutting and other human management activities means that any change in management practices, in conjunction with the impacts of larger-scale factors such as climate change, can greatly affect the structure and functions of these habitats (Joyce and Wade 1998, Tallowin and Jefferson 1999, Joyce 2014, Joyce et al. 2016). In some areas, <1% of the estimated original extent of wet grasslands remains due to the dual impacts of agricultural intensification or abandonment (cessation of cutting) (Luoto et al. 2003, Joyce 2014).

An earlier field study, which investigated the impact of nutrient addition on plant growth and production and soil properties in two wet grasslands with peat or mineral soils, noted accelerated nutrient fluxes in both wet grasslands, but especially in the one with peat soil (Picek et al. 2008, Edwards 2015, Edwards et al. 2015). In addition, nutrient levels interacted with site hydrology to affect plant growth and production, which likely allowed for the co-existence of common plant species belonging to different plant functional types (Edwards and Čížková 2020). Therefore, several environmental factors, acting both singly and interactively, affect the ecological functioning of these wet grasslands; however, the importance of these different factors could not be easily disentangled in the field. Determining the importance of these various environmental factors and how they may interact is quite necessary to properly manage these important ecological systems.

In addition, while various soil properties, such as microbial nutrient contents, were measured in the field (Picek et al. 2008), soil microbial community composition was not included in previous studies. We therefore established a mesocosm in order to investigate the impact of multiple environmental drivers on soil microbial community structure under more controlled conditions than could be achieved in the field.

The objectives of this study were to determine the effect of (1) changing environmental conditions ( i.e., context dependence), namely differences in soil type, hydrology, and nutrient levels, and (2) the presence or absence of plants on soil microbial community structure. Based on the results from our previous field study as well as literature sources, we hypothesized that: (1) soil microbial composition would be most impacted by nutrient addition, with the other environmental factors, water level and soil type, having secondary impacts; (2) both single factors and their interactions will significantly influence microbial community structure; (3) there will be a context-dependent shift in the dominance of r- and K-strategists, with r-strategists favored in nutrient-richer, moist but not saturated conditions; and (4) since the field study showed that the wet grasslands are plant-dominated systems (Picek et al. 2008), plant growth promoting microbes (i.e. diazotrophs, heterotrophic bacteria) will be the dominant groups in the different treatment combinations.

## Methods

### Mesocosm establishment

**Figure 1.**
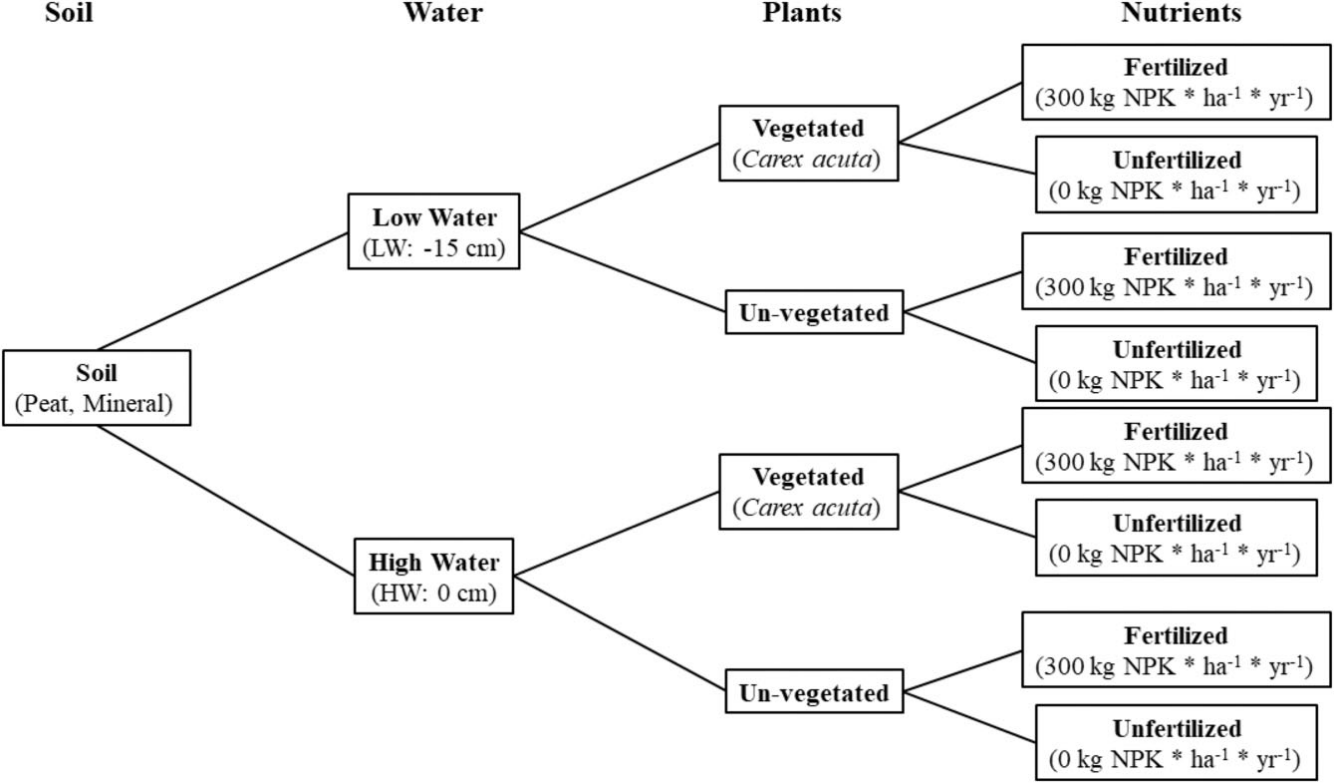
Schematic of the full-factorial experimental treatment design used in our study. Treatments consisted of two soil types (Soil: peat, mineral), two water levels (low = 15 cm below soil level, high = at soil surface), two nutrient levels (unfertilized, fertilized = 300 kg NPK*ha^−1^ * yr^−1^) and vegetated or un-vegetated conditions. Treatment combinations were then randomly applied to particular basins. See text for details.

A mesocosm was established at the University of South Bohemia in July 2009 to help disentangle the effects of soil type, water level, and fertilization on PSM interactions in wet grasslands using a full factorial design (Fig. 1). We used plants of *Carex acuta*, a common plant species of wet grasslands in Central Europe. Although *C. acuta* is considered to be a conservative, stress-tolerator species, it is co-dominant with more competitive species such as *Glyceria maxima* under a range of nutrient and water level conditions (Edwards and Čížková 2020). *Carex acuta* plants, consisting of multi-stemmed ramets, were collected from the field in April 2009. The ramets were divided into separate shoots with attached roots, which were planted in 0.5 L containers filled with sand and placed into a shallow (15 cm depth) basin with water to 7 cm (providing saturated, but not flooded conditions) to recover from the removal and transplanting processes. Following this two-week acclimation period, the plants were divided by height (small: <5 cm; medium: >5 but <8 cm; tall: >8 cm), and four healthy-looking plants (one small, two medium, and one tall) were planted into pots (40 × 40 × 35 cm; L x W x D), which were randomly distributed to the different experimental treatment combinations (Fig. 1). The pots were placed into basins (180 × 110 × 45 cm; L x W x D), located in an outdoor enclosure at the University of South Bohemia, in order to more easily control water and nutrient levels. Each pot contained either a mineral soil or peat soil. The mineral soil consisted of a cambisol taken from a nearby meadow, while the peat soil was developed by mixing the mineral soil with peat amendments. Initial levels of basic soil parameters are given in Supplementary Table S1. Each basin was subjected to one of two water level treatments: either saturated conditions, in which the water level was maintained at the soil surface (HW treatment), or a drier treatment, in which the water level was kept 15 cm below the soil surface (low-water treatment). Water was added to the basins when needed. Two holes were drilled into the sides of each basin at the correct height, depending on the particular water level treatment randomly assigned to each basin, to prevent water levels above the desired levels. The nutrient treatment consisted of no added nutrients (unfertilized treatment) or fertilized (nutrient addition, 300 kg NPK * ha^−1^ * yr^−1^) using a mineral NPK fertilizer (Lovofert 15–15–15 NPK, Lovochemie, a.s.). This NPK solution was applied in two half doses (mid-May and early July) to selected pots to simulate the normal procedures of local farmers. In addition, a micronutrient solution (Ruakura micronutrient solution) was added to all pots with plants to ensure that micronutrients did not become limiting. A treatment combination (soil, water, and nutrient) was assigned randomly to each basin, with there being two replicate basins for each treatment combination, for a total of 16 basins. Each basin contained ten pots, six with plants while the other four were un-vegetated, but all subjected to the same treatment combination. The greater number of vegetated pots was due to the need for more plant material to carry out other plant-based studies. In addition, the number of un-vegetated pots per basin was considered to be sufficient for the purposes of this study, the determination and comparison of soil microbial community structure. The treatments were applied over five years, until the end of the experiment in 2013. Data from five years of treatments (the 2013 growing season) were used for this study.

### Sample collections

Soil and above- and belowground plant parts were sampled four times during 2013 to determine seasonal effects on above- and belowground plant biomass, soil physico-chemical properties [pH, soluble organic carbon (SOC), and total soluble nitrogen (TSN)], and microbial community composition: once before the growing season (March) and three times during the growing season—spring (May), summer (July), and autumn (September). On each sampling date, two 10 × 10 cm plots were placed into selected pots from which aboveground material was harvested. Then cores of 7 cm diameter (15 cm depth) were taken within each of these plots. The holes were re-filled with the respective soil after coring. Aboveground samples were separated into live and dead components for *Carex* and then any other species. All separated fractions were placed into labeled paper bags, dried at 65°C, and weighed. Belowground samples were carefully cleaned, separated into live and dead components, and each of these further divided into roots and rhizomes. The fractions were also placed into labeled paper bags, dried at 65°C for at least 48 h, and weighed. Net aboveground and belowground primary production (NAPP and NBPP, respectively) were calculated from these biomass data using the methods of Picek et al. (2008) and Edwards and Čížková (2020).

Soil cores (4 cm diameter; 15 cm depth) were taken from two randomly selected vegetated and un-vegetated pots in each basin for a total of four samples from each treatment combination. The cores from the vegetated pots were taken in other corners of the pots unaffected by the plant sampling. These samples were then used to determine soil physico-chemical parameters and microbial community composition.

### Molecular analyses

The Power Soil DNA Isolation Kit (MoBio Laboratories Inc., Carlsbad, CA, USA) was used for the isolation of genomic DNA from soil according to the manufacturer’s instructions with some modifications. A mini Bead-Beater (Bio-Spec Products, Inc.) was used at a speed of 6 ms^−1^ for 45 s for better disruption of cell walls. DNA was stored in 1.5-mL Eppendorf microtubes in a freezer (-20°C) until analysis. The quality of the extracted DNA was verified by electrophoresis (1% w/v, 8 V cm^−1^, and 45 min). Total DNA was quantified using SybrGreen fluorescence methodology (Leininger et al. 2006).

### qPCR analysis

Quantification of bacterial and fungal SSU rRNA genes was performed using the FastStart SybrGREEN Roche® Supermix and Step One system (Life Technologies, USA). Each reaction mixture (20 µL) contained 2 µL DNA template (∼1–2 ng DNA), 1 µL each primer (0.5 pmol µL−1 each, final concentration), 6 µL dH_2_O, 10 µL FastStart SybrGREEN Roche® Supermix (Roche, France), and 1 µL BSA (Fermentas, 20 mg µL^−1^). Initial denaturation (3 min, 95°C) was followed by 30 cycles of 30 s at 95°C, 30 s at 62°C, 15 s at 72°C, and completed by fluorescence data acquisition at 80°C used for target quantification. Product specificity was confirmed by melting point analysis (52–95°C with a plate read every 0.5°C), and amplicon size was verified with agarose gel electrophoresis. Bacterial standards consisted of a dilution series (ranging from 10^1^ to 10^9^ gene copies µL^−1^) of a known amount of plasmid with a cloned amplicon from *Escherichia coli*DNA by using the SSU gene-specific primers 341F/534R (Muyzer et al. 1993). R^2^ values for the standard curves were >0.95. Slope values were >3.38 giving an estimated amplification efficiency of >92%.

The qPCR conditions for fungal quantification were as follows: initial denaturation (10 min, 95°C), followed by 40 cycles of 1 min at 95°C, 1 min at 56°C, and 1 min at 72°C, and completed by fluorescence data acquisition at 72°C used for target quantification. Fungal standards consisted of a dilution series (ranging from 10^1^ to 10^7^ gene copies µL^−1^) of a known amount of plasmid with a cloned amplicon from genomic *Aspergillus niger* DNA by using the SSU gene-specific primers nu-SSU-0817–5′ and nu-SSU1196-3′44 (Borneman and Hartin 2000). R^2^ values for the fungal standard curves were >0.99. The slope was between 3.34 and 3.53, giving an estimated amplification efficiency from 93% to 95%, respectively.

Detection limits (i.e. lowest standard concentration that is significantly different from the non-template controls) were <100 gene copies * µL^−1^ for each of the genes. Samples, standards, and non-template controls were run in triplicate. Enhancers (BSA) were added to the PCR mixture to deal with potential inhibition during PCR. Also, several dilutions (10x, 100x, and 1000x) for representative samples were tested beforehand to see the dilution effect on Ct values.

Sequencing of the prokaryotic community was conducted using the PCR primers 515F/806R to target the V4 region of the 16S rRNA gene (Caporaso et al. 2010, 2011). The PCR mixture contained 13 µL MO BIO PCR-grade water, 10 µL 5 PRIME Hot Master Mix, 0.5 µL each of the forward and reverse primers (0.2 µM final concentration), and 1 µL of genomic DNA. Samples were kept at 94°C for 3 min to denature the DNA, with amplification proceeding for 35 cycles at 94°C for 45 s, 50°C for 60 s, and 72°C for 90 s; a final extension of 10 min at 72°C was added to ensure complete amplification. The reverse primer contained a 12-base error-correcting Golay barcode to facilitate multiplexing. Each sample was amplified in triplicate, combined, and quantified using Invitrogen PicoGreen® and a plate reader, and equal amounts of DNA from each amplicon were pooled into a single 1.5-mL microcentrifuge tube. Cleaned amplicons were quantified using PicoGreen dsDNA reagent in 10 mM Tris buffer (pH 8.0). A composite sample for sequencing was created by combining equimolar ratios of amplicons from the individual samples and cleaned using the Ultra Clean® htp 96-well PCR clean-up kit (MO BIO Laboratories). Purity and concentration of the samples were estimated by spectrophotometry. Amplicons of 250 bp were sequenced pair-end (150 × 150 cycles) on the Illumina MiSeq platform (Argonne National Laboratory, IL, USA).

Bacterial raw pair-end reads (150 bp) were joined using ea-utils to obtain reads of cca 250 bp length (Aronesty 2013). Quality filtering of reads was applied as described previously (Caporaso et al. 2011). Reads were truncated at a phred quality threshold of 20. Reads that contained ambiguities and reads whose barcode did not match an expected barcode sequence were discarded. Reads were assigned to operational taxonomic units (zOTUs = zero radius OTU) using the Uparse pipeline (Edgar 2013). Taxonomy was assigned to each read by accepting the ARB Silva v.132 taxonomy string of the best megablast matching the ARB Silva v.132 sequence. This resulted in an OTU table showing the relative distribution of zOTUs in each sample.

The relative abundance ( = proportion) of each zOTU in each sample was then multiplied by the number of known copies of the 16S rRNA gene for that sample, as determined by qPCR (Bárta et al. 2017). This re-calculation resulted in producing absolute abundances for each zOTU, which were then used in subsequent statistical analyses (see below).

Raw sequencing data were deposited on the ENA (European nucleotide archive) server under the study PRJEB54693.

### r/K analyses

From the normalized zOTU table, we were able to calculate the community average genome SSU copy number (ACN) in each sample for the selected archaea and bacterial families (Thompson et al. 2017). The ACN was calculated from the raw and normalized zOTU table (the first step in the PICRUSt pipeline; Langille et al. 2013). SSU gene copies range from 1 to 15 in microbial genomes. Copiotrophic (r-strategists) microbes are assumed to have more SSU gene copies in the genome; therefore, a higher average ACN shows the higher proportion of copiotrophic taxa in the microbial community.

### Data analyses

The effects after five years of experimental treatments on soil pH, SOC, and TSN and net above- and belowground primary production (NAPP and NBPP, respectively) were tested by ANOVA following natural logarithm or square root transformations when needed to achieve data normality and homogeneous variances. The ANOVAs were run using SYSTAT v 11. NAPP and NBPP were estimated based on the harvested plant biomass in the four sampling times (Edwards 2015, Edwards and Čížková 2020).

The number of gene copies per ng DNA (based on qPCR) was used to calculate the bacteria-to-fungi (B/F) ratio. The sequenced archaea and bacteria had to meet several criteria in order to be included in subsequent analyses. For the analysis of the prokaryotic microbiome, we selected only the most abundant phyla (>1% of total bacterial zOTUs), classes (>3% of the zOTUs for a particular selected bacterial phylum), and families (>1% for a selected phylum) (Supplementary Table S2). Univariate and multivariate methods were then used to determine the effect of the experimental treatments (plant presence/absence; soil type; nutrient addition; water level) on the separate datasets of the archaea and bacterial community structures (two datasets of, first, the selected bacterial phyla plus the Proteobacteria classes and, second, the selected families). When needed, natural logarithm or square root transformations of the data were used to meet the criteria for normality and homogeneous variances.

Differences in the soil prokaryotic microbiome among the different experimental factors were tested by running principal components analyses (PCA) and redundancy analyses (RDA) separately on the archaea classes and the selected bacterial phyla in PcOrd, v. 7.0 (McCune and Mefford 2018). Permanovas were run to determine the effects of the different environmental factors, as well as the two-way interactions, on microbial composition using the "adonis" program in the VEGAN package in R v. 4.0.0 (Oksanen et al. 2020, R Core Team 2020) with 999 permutations.

Generalized linear mixed models (GLMM) were used to determine the impact of the environmental factors on individual selected phyla, classes, and families of the archaea and bacterial zOTUs. The GLMM analyses were conducted in R v. 4.0.5 (R Core Team 2021) with the nlme package (Pinheiro et al. 2016). The experimental treatments (plants, soil, water, and nutrients) were the fixed factors, while month was a random factor. All of the mixed-effect models were run following the procedure outlined in Zuur et al. (2009). The best model for each dependent variable tested was chosen based on a comparison of Akaike information criteria (AIC) scores (Akaike 1987).

Differential analyses were conducted on the natural logarithm-transformed total zOTU counts of the selected archaea and bacterial families (Chialva et al. 2020). Independent analyses were performed on the environmental treatment factors (soil, water level, nutrient addition), as well as plant presence or absence, to show the treatment conditions preferred by the selected families. The analyses were conducted in SYSTAT v. 11.

Repeated measures ANOVA in SYSTAT v. 11 found few significant seasonal effects in the 2013 dataset with the exception of Actinobacteria and Chlorobi. The absolute number of bacterial zOTUs in those two phyla was significantly lower in March compared to the May, July, and October samples. Statistical analyses were therefore conducted on datasets either with or without the March data. Since the results were similar for both datasets, we report the results of the full dataset, which included all seasons.

Lastly, the effects of the experimental treatments on the differences in the distribution of r- and K-strategists were tested by Chi-square (χ^2^) analysis in SYSTAT v. 11.

## Results

### Soil physico-chemical characteristics

The peat soil was more acidic and contained more C and N than the mineral soil, both initially and after five years of experimental treatments (Supplementary Table S1). In 2013, the pH of both soils was significantly greater in the high water (HW), un-vegetated treatments (significant water level * plant interaction, F_1, 80_ = 3.25, *P* = 0.05), while SOC and TSN levels were significantly greater in fertilized peat soil, but only SOC had a significant soil * nutrient interaction term (F_1, 80_ = 4.01, *P* = 0.048). TSN was significantly greater in HW, un-vegetated samples (water level * plant interaction, F_1, 80_ = 24.48, *P* < 0.001).

SOC and TSN levels also differed over time, with SOC levels being significantly higher in September (L_16, 18_ = 25.66, *P* < 0.001). Maximum TSN levels occurred in March, with these being significantly greater than the September levels (L_16, 18_ = 72.658, *P* < 0.001).

### Plant production

Aboveground plant production (NAPP) ranged from a minimum of 439.75 gDW m^−2^ yr^−1^ in the mineral, HW, unfertilized treatment to a maximum of 1251.25 gDW m^−2^ yr^−1^ in the peat, low-water, fertilized treatment (Supplementary Table S1b). Only nutrient addition significantly affected NAPP (F_1, 9_ = 13.42, *P* = 0.005), with fertilization having a positive impact on NAPP, while there was a weakly negative water level effect (F_1, 9_ = 4.944, *P* = 0.053). Soil type and nutrient addition, both singly and their interaction, significantly affected NBPP (soil: F_1, 8_ = 21.609, *P* = 0.002; nutrients: F_1, 8_ = 34.016, *P* < 0.001; soil * nutrient interaction: F_1, 8_ = 7.449, *P* = 0.026) with NBPP being greater in peat and fertilized conditions (Supplementary Table S1b). Overall, changes in both NAPP and NBPP were more apparent in the mineral soil.

### Microbial community structure

#### B/F ratio

**Figure 2.**
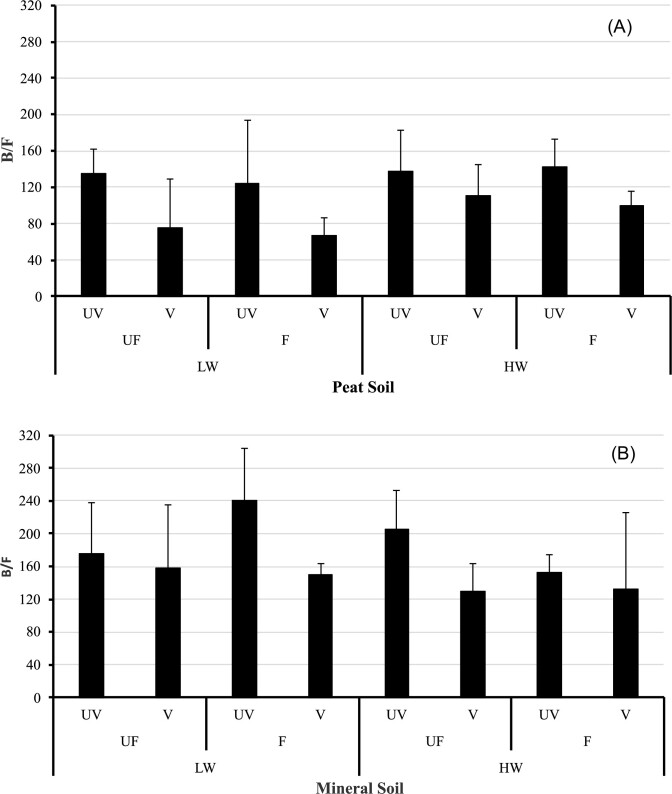
Mean (± s.d., *n* = 8) bacteria-to-fungi (B/F) ratio in (A) peat; (B) mineral soils. Treatments: water level: low (LW = 15 cm below soil level), high (HW, at soil surface); Fertilization: UF (unfertilized), F (300 kg NPK*ha^−1^ * yr^−1^); Plants: UV (un-vegetated), V (vegetated).

Bacteria dominated the mesocosm system being at least two orders of magnitude greater than fungal abundance, with the sample B/F ratio ranging from 38 to >300 (Fig. 2). There were significantly more OTUs assigned to bacteria than fungi in the mineral soil, while fungal OTUs were more numerous in peat, although these were still fewer than bacteria (significant soil effect, t = 26.106, *P* < 0.001). Fungi were further supported in vegetated soil (significant plant effect, t = 16.870, *P* < 0.001), which was more apparent in the peat soil under low-water conditions (significant soil * water level effect; t = 9.655, *P* = 0.002).

#### Archaeal and bacterial microbiome

The number of obtained sequences ranged from 7.77 * 10^8^ in March to 1.18 * 10^9^ in July. Of these, archaea accounted for only 1.2%–1.3% of the sequences, neither of which differed seasonally.

Sequencing identified three archaea phyla, nine classes, and 13 families, of which seven classes and eight families were used for further analyses. Bacterial OTUs were classified into 58 phyla, of which 11 were selected for further analyses (Supplementary Table S2). In most samples, Proteobacteria accounted for more than a third of all the bacterial sequences (range 29.8%–51.7%), followed by Acidobacteria (6.4%–20.7%), Bacteroidetes (3.6%–20.9%), Actinobacteria (5.2%–11.0%), and Verrucomicrobia (7.4%–12.0%). The other selected phyla accounted for <5% of the sequences.

### Impact of the treatment factors on microbiome structure and abundance

**Figure 3.**
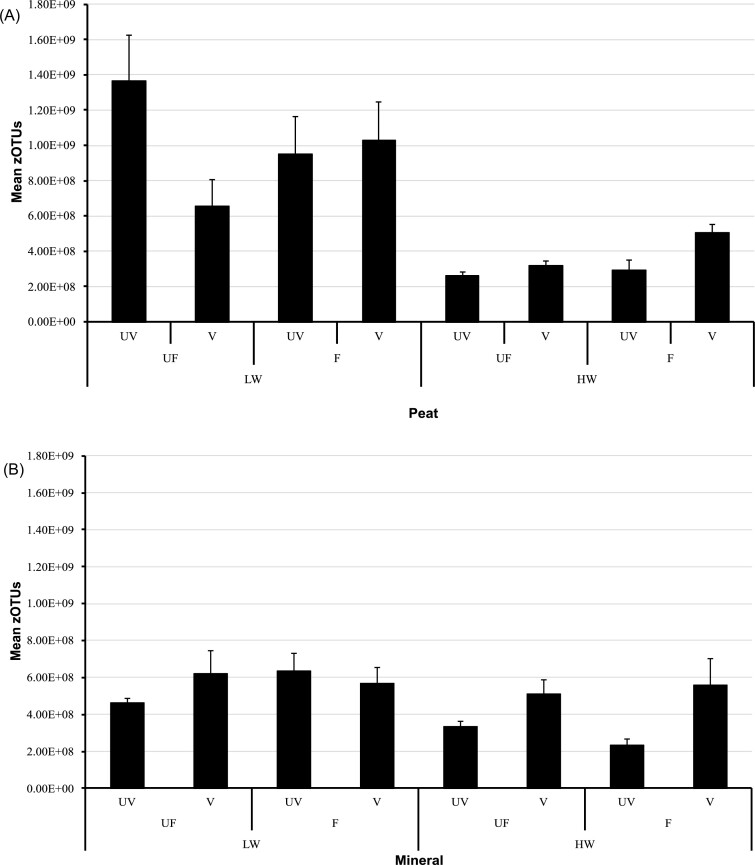
Total bacterial absolute abundance (means ± 1 SE; *n* = 8; zOTU g^−1^ dw soil) in the different treatment combinations in (A) peat and (B) mineral soils. Treatments: water level: LW = low-water (15 cm below soil surface); HW = high water (saturated, water level maintained at the soil surface); nutrients: UF = unfertilized (0 kg NPK ha^−1^ yr^−1^); F = fertilized (300 kg NPK ha^−1^ yr^−1^); plants: UV = un-vegetated; V = vegetated.

Soil type, water level, plant presence or absence, and their interactions, significantly affected total microbial abundances (*P* < 0.05; GLMM analysis), while nutrient supply had little impact. Total microbial abundance was the greatest in the peat, low-water, unfertilized, un-vegetated treatment, but was also greater in vegetated, HW conditions (significant water * plant interaction, *P* < 0.001), especially in the mineral soil (Fig. 3).

**Figure 4.**
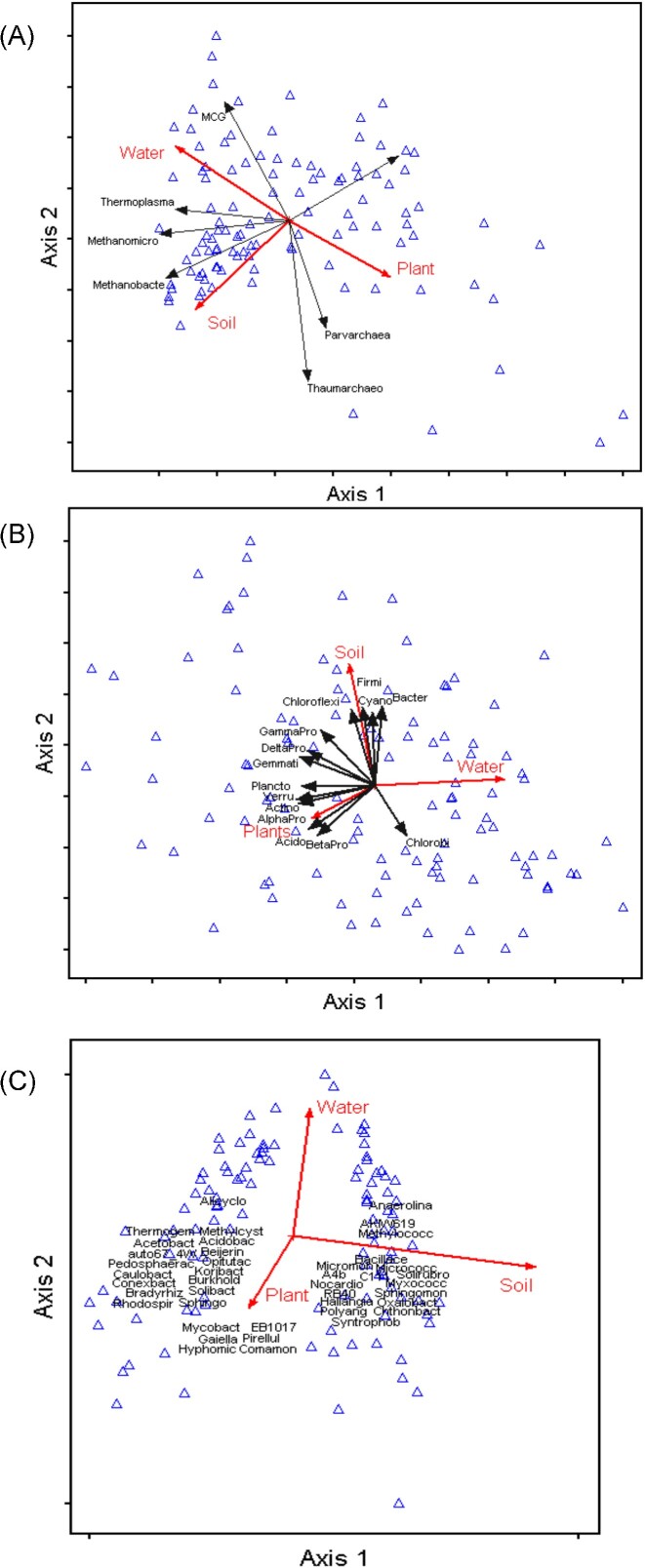
Axes 1 and 2 of RDAs for (A) Archaea classes, (B) selected bacteria phyla, including the alpha, beta, delta, and gamma Proteobacteria classes, and (C) selected bacterial families from OTU data. Blue triangles = samples. Black arrows = microbial taxa. Only significant experimental factors (red arrows) shown. Acronyms: Archaea: MethBact = Methanobacteria; MethMicr = Methanomicrobia; Parva = Parvarchaeota; Thaum = Thaumarchaeota; Thermo = Thermoplasmata. Bacteria phyla: Acido = Acidobacteria; Actino = Actinobacteria; Bacter = Bacteroidetes; Cyano = Cyanobacteria; Firmi = Firmicutes; Gemmati = Gemmatimonadetes; Plancto = Planctomycetes; Verru = Verrucomicrobia; AlphaPro = Alphaproteobacteria; BetaPro = Betaproteobacteria; DeltaPro = Deltaproteobacteria; GammaPro = Gammaproteobacteria. Bacteria families: Acetobact = Acetobacteraceae; Acidobact = Acidobacteriaceae; Alicyclo = Alicyclobacillaceae; Anaerolina = Anaerolinaceae; Bacillace = Bacillaceae; Beijerin = Beijerinckiaceae; Bradyrhiz = Bradyrhizobiaceae; Burkhold = Burkholderiaceae; Caulobact = Caulobacteraceae; Chthonbact = Chthoniobacteraceae; Comamon = Comamonadaceae; Conexbact = Conexibacteraceae; Gaiella = Gaiellaceae; Haliangia = Haliangiaceae; Hyphomic = Hyphomicrobiaceae; Koribact = Koribacteraceae; Methylococc = Methylococcaceae; Methylcyst = Methylocystaceae; Micrococc = Micrococcaceae; Micromon = Micromonosporaceae; Mycobact = Mycobacteriaceae; Myxococc = Myxococcaceae; Nocardio = Nocardiaceae; Opitutac = Opitutaceae; Oxalobact = Oxalobacteraceae; Pedosphaerac = Pedosphaeraceae; Pirellul = Pirellulaceae; Polyang = Polyangiaceae; Rhodospir = Rhodospirillaceae; Solibact = Solibacteraceae; Solirubro = Solirubrobacteraceae; Sphingo = Sphingobacteriaceae; Sphingomon = Sphingomonadaceae; Syntrophob = Syntrophobacteraceae; Thermogem = Thermogemmatisporaceae.

For the archaea, Methanobacteria, Methanomicrobia, and Thermoplasmata (all Euryarchaeota), which preferred growing in un-vegetated, saturated (HW) mineral soil (significant soil * water * plant interaction: F_1, 66_ = 17.40; *P* < 0.001; Supplementary Fig. S1a), were separated from archaea classes associated more with the peat soil (MBGA and Thaumarcheota—both Crenarchaeota—and Parvarchaea) along axis 1 of the PCA (Supplementary Fig. S2a, b). Water level differences further separated the mineral-preferring group of Euryarchaeota along axis 1, as well as those classes associated more with peat soil (significant soil * water interaction, *P* = 0.001) along axis 2 of the PCA (Supplementary Fig. S2a, b) (RDA: soil r = 0.367 and 0.481 for axes 1 and 2, respectively; water r = 0.371 and 0.309 for axes 1 and 2, respectively; Fig. 4A). Plant presence or absence was an important, though secondary, factor related to axis 1 (RDA: plant r = 0.326), while both nutrient addition and plant presence were the most important factors related to axis 3 (PCA explained variance = 17.20%; RDA r = 0.272 and 0.232 for nutrients and plants, respectively).

**Figure 5.**
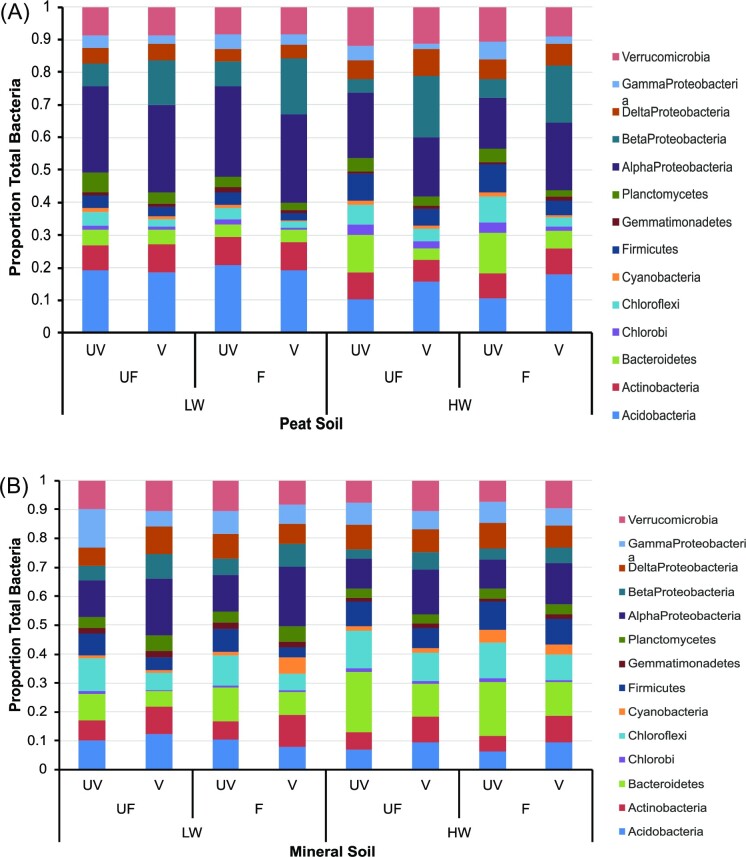
Relative abundance of selected bacteria phyla (average from four sampling dates in March, May, July, and September 2013; *n* = 8) and the alpha-, beta-, delta-, and gamma-Proteobacteria classes found in either peat soil (A) or mineral soil (B) under different treatment conditions in a mesocosm experiment. Treatments: water level: LW = Low (15 cm below soil surface), HW = high (saturated, water level maintained at the soil surface); fertilization: UF = unfertilized (0 kg NPK ha^−1^ yr^−1^), F = fertilized (300 kg NPK ha^−1^ yr^−1^); plants: UV = un-vegetated, V = vegetated.

Both soil type and water level were the main factors affecting bacterial abundance at both the phylum and family levels. At the phylum level, bacteria divided into two groups based on their particular environmental preferences. The relative abundances of Bacteroidetes, Chloroflexi, and Firmicutes (Fig. 5) were significantly greater in saturated, un-vegetated, mineral soils (*P* < 0.05), in contrast to the Acidobacteria, Actinobacteria, and alpha- and beta-Proteobacteria groups (*P* < 0.001). This separation is also noted in the respective PCA and RDA of the absolute abundances of the selected bacterial phyla. Water level (RDA r = 0.513; Fig. 4B) was the main factor separating bacterial phyla along axis 1 of the PCA, with those preferring saturated conditions on the right side of axis 1 (Supplementary Fig. S3). Phyla that preferred mineral soil separated from those associated with the peat soil along the second axis (RDA r = 0.441; Fig. 4B; Supplementary Fig. S3).

Bacterial phyla abundances changed little between months, except for Actinobacteria (*P* = 0.021), Chlorobi (*P* = 0.004), and delta-Proteobacteria (*P* < 0.001). Actinobacteria had a significantly greater proportion of the total selected phyla during the growing season (lowest proportions in March), while the other two had significantly higher proportions in March and September.

**Figure 6.**
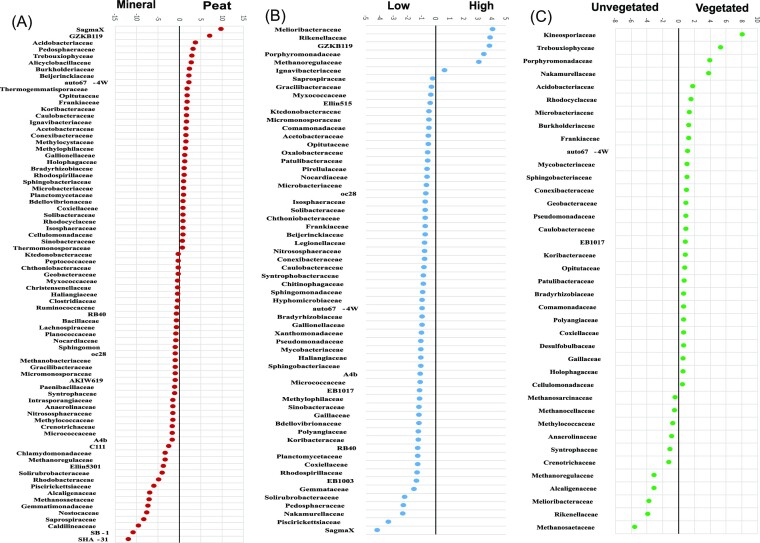
Dot plots of significant differentially abundant families between the mineral and peat soils (A); preference for either the low (15 cm below the soil surface) or high (saturated) water level treatments (B); and preference for either the vegetated or un-vegetated treatments (C). Only families that had a significant preference (*t*-test; *P* < 0.05) are shown. Analyses were conducted on natural logarithm-transformed abundance data.

The first two axes of the selected bacterial family PCA (Supplementary Fig. S4) explained 58.44% of the variance, being almost equal in their effect. Conversely to the phylum analysis, soil type was the main factor related to axis 1 (RDA r = 0.903; Fig. 4C) with the Chloroflexi, Firmicutes, and delta-Proteobacteria families preferentially growing in mineral soil, along with the methanogenic archaea and Nitrososphaeraceae (Crenarchaeota). On the contrary, many of the Acidobacteria, Planctomycetes, Verrucomicrobia, and the alpha- and beta-Proteobacteria families preferred peat soil (Fig. 6A).

Water level was the factor most related to axis 2 (RDA r = 0.555; Fig. 4C; Supplementary Fig. S4). Most of the families preferentially grew in drier conditions (low-water treatment; Fig. 6B) with these forming a group in the upper part of the RDA graph (Fig. 4C). Exceptions to this included several of the methanogenic archaea (Methanoregulaceae, Methanospirillaceae), the two Chlorobi families, some of the Bacteroidetes families, and Syntrophaceae (delta-Proteobacteria) (Fig. 6B).

Plant presence was an important but secondary factor associated with axis 1 of the bacterial phyla analysis (RDA r = 0.338), and was also the main effect related to axis 3 of the bacterial family analysis (PCA = 7.58%; RDA r = 0.430). Most of the selected bacterial families were significantly more abundant in vegetated samples, with the exception of several Chlorobi, Chloroflexi and Firmicutes families as well as the methanogenic archaea (Fig. 6C).

### r/K analysis

We were able to determine the strategy for 104 of the 107 selected families, with 33 identified as r-strategists and the other 71 being K-strategists (Table 1; Supplementary Table S2). K-strategists significantly preferred growing in the peat soil under the low-water treatment (significant soil * water level interaction), but in the HW treatment in mineral soil (χ^2^ = 11.682; *P* = 0.009). There was also a significant water * plant interaction (χ^2^ = 8.698; *P* = 0.034), in which 42 of 52 families were more abundant in the vegetated, low-water treatment, with 11 designated as r-strategists and the remaining 31 as K-strategists.

**Table 1.** Results of general linear mixed model (GLMM) analyses on the absolute abundances (OTUs) of total selected microbes (archaea + bacteria) for selected microbial taxa showing the main and two- and three- factor interactive effects of soil (S), water level (W), nutrient addition (F = Fert), and plants (P).

(A) Archaea families (abundance)														
	Soil	Water	Fert	Plants	S*W	S*F	S*P	W*F	W*P	F*P	SWF	SWP	SFP	WFP
SAGMA X	P>M ***	L>H ***			***				**		*			
Nitrososphaeraceae	M>P ***	L>H ***					**		**					
Methanobacteriaceae	M>P ***			UV>V **							*	**		
Methanocellaceae				UV>V***			**			*	**	**		
Methanoregulaceae	M>P ***	H>L**		UV>V***			**		*					
Methanosaetaceae	M>P ***	H>L**		UV>V***			***					**		
Methanosarcinaceae		H>L**		UV>V**		*				*	*			
Methanomassilii-coccaceae	M>P ***	H>L***	UF>F***	UV>V***		*		**	**	**	**			**
(B) Selected bacteria phyla / Proteobacteria classes (abundance)														
Acidobacteria	P>M***	L>H***		V>UV***	***				***	*				
Actinobacteria		L>H***		V>UV***	**	+	***		***	**				
Bacteroidetes	M>P***			UV>V**	***					+				
Chlorobi	P>M***			UV>V***			*		*					
Chloroflexi	M>P***	L>H***		UV>V**			**		*					
Cyanobacteria	M>P**				***		*		*					
Firmicutes	M>P***			V>UV+			*		**					
Gemmatimonadetes	M>P***	L>H***		V>UV***		+			***					
Planctomycetes		L>H***	UF>F*		**		***		*					
Alphaproteobacteria	P>M***	L>H***		V>UV***	**		*		***					
Betaproteobacteria	P>M***	L>H***		V>UV***					***			+		
Deltaproteobacteria	M>P***	L>H***		V>UV**					**			*		
Gammaproteobacteria	M>P***	L>H***		UV>V*	***		**		+					
Verrucomicrobia		L>H***		V>UV**			**		**					
(C) Functional g (abundance and proportion of total selected microbes)														
Methanogens	M>P	H>L		UV>V										
Abundance	***	**		***			**		***		*	**		
Proportion	***	***		***			*				**			
Methanotrophs	M>P	H>L		UV>V										
Abundance	***	***		*	*		**		*			**		
Proportion	***	***		***										
OM Degraders	P>M	L>H		V>UV										
Abundance	***	***		***	***		*		***	+				
Proportion	***	***		***	***				+					
Diazotrophs	M>P	L>H		V>UV										
Abundance	***	***		*	***		*		***					
Proportion					***									
Nitrifiers	M>P	L>H	F>UF	UV>V										
Abundance	***	***	**	*	***	*	***	***	***		*	**		*
Proportion	***	***			***	*	***	**	**		*			
FRB	M>P	H>L		UV>V										
Abundance	***	***		+			**		*		*			
Proportion				*										
(D) r/k strategists (abundance)														
r/k					k>r**				k>r*					

(A) archaea families; (B) bacteria phyla, including selected Proteobacteria classes; (C) functional groups (absolute abundance and proportion of total selected microbes); (D) r/k strategists. Factors: Soil (*P* = peat; M = mineral); Water (L = low (15 cm below soil surface); H = high (saturated, water level maintained at the soil surface); Nutrients (Fert) (UF = unfertilized (0 kg NPK ha^−1^ yr^−1^); F = fertilized (300 kg NPK ha^−1^ yr^−1^); plants (UV = un-vegetated; V = vegetated). *P* values: * = < 0.05; ** < 0.01; *** < 0.001; + < 0.10.

## Discussion

### B/F ratio

Bacteria were the dominant microbial group in our experimental system, as noted by the B/F ratio (Fig. 2). Such low numbers of fungi are a common feature of many wetland habitats (Gutknecht et al. 2006). Still, based on the B/F results, the proportion of fungi was significantly greater in peat soil under low-water conditions. This treatment had a greater, though not significant, amount of C (measured as SOC) in relation to the other treatments (soil * water interaction, *P* = 0.062). This finding is in agreement with other studies, which showed that increased fungal numbers (lower B/F) were related to greater C storage (Strickland and Rousk 2010, Malik et al. 2016). However, the B/F ratio is a simple index, the use and interpretation of which must be done with great caution (Fierer 2017).

### Treatment effects

In our study, the archaea accounted for <2% of total OTUs, which is similar to the proportion noted by Bates et al. (2011). Likewise, Proteobacteria was the most abundant phylum in our study, followed by Acidobacteria, Actinobacteria, Bacteroidetes, and Verrucomicrobia. These tend to be common phyla in many bacterial microbiome studies (e.g. see the tables in Hawkes et al. 2007 and Mendes et al. 2013, Delgado-Baquerizo et al. 2018).

Soil type, water level, and plant presence significantly influenced soil microbial structure, both singly and interactively; however, nutrient addition had little or no impact. Therefore, our results only partially support our first hypothesis. The lack of a significant nutrient effect differs from other studies (e.g. Marschner et al. 2003, Jangid et al. 2008, Zhang et al. 2017, Wang et al. 2018, Tahovská et al. 2020), but is similar to the results of short-term fertilization studies (Buyer and Kaufman 1996, Crecchio et al. 2001). The lack of a significant nutrient effect may be due to us recording the effects after only five years of experimental treatments, which may be too short of a time for changes to become apparent (Marschner et al. 2003).

In addition, application of inorganic fertilizers, as done in our study, promotes indirect effects of nutrient addition by increasing plant biomass, while direct application of organic matter can lead to a significant increase in bacterial biomass as well as favoring copiotrophs over oligotrophic microbes (Hu et al. 1999, Marschner et al. 2003). Nutrient addition significantly increased both NAPP and NBPP, which would result in a greater quantity of higher-quality plant litter available for decomposition (Saggar et al. 1997; Cheng et al. 2020), as well as possibly greater root exudate inputs (Neumann et al. 2014). The addition of greater quantities of higher-quality plant inputs likely led to the significant increase in SOC in the vegetated samples, while the significant reduction of TSN when plants were present was likely due to plant uptake resulting from increased plant growth.

In our study, most of the microbes preferentially grew in low-water conditions when plants were present, while methanogens (archaea—class Methanomicrobia) and other strict anaerobic bacteria, like several Bacteroidetes families (GZKB119, Porphyromonadaceae, notably *Paludibacter* and Rikenellaceae; Ueki et al. 2006; Nakasaki et al. 2020), and the iron-reducing members of the Chlorobi families (Ignavibacteriaceae and Melioribacteraceae; Iinu et al. 2010; Podoskoroskaya et al. 2013, Fortney et al. 2016), were significantly enhanced in the un-vegetated HW treatment samples. Thus, our water level treatment separated aerobic microbes from those able to tolerate low oxygen or anaerobic conditions, similar to other studies that noted differential soil microbial structure resulting from changed hydrologic patterns (Unger et al. 2009, Wang et al. 2015; Gonzalez et al. 2016, Chialva et al. 2020).

The microbial community structure differed depending on the peat content of the soil, with particular phyla preferentially growing in either the mineral or peat soil (Lundberg et al. 2012). For example, most families of the Chloroflexi, Firmicutes, delta-Proteobacteria, the Euryarchaeota, and the Nitrososphaeraceae (Crenarchaeota) were associated with the mineral soil. In contrast, the Acidobacteria, Planctomycetes, Verrucomicrobia, and alpha- and beta-Proteobacteria were more indicative of the peat soil, as were several known extremophile families, such as SAGMA-X (Crenarchaeota), which tolerate highly acidic conditions (Takai et al. 2001). Microbial community structure varied more in the peat soil, as shown by the PCAs for the archaea classes and bacterial phyla and families (Supplementary Fig. S2–4), in which the samples and centroids of microbial abundance in the mineral soil formed a tighter cluster compared to the greater spread of the peat soil samples. This is likely related to soil pH (Hansel et al. 2008), the range of which was much narrower for the mineral soil treatments (5.84–6.15) compared to the peat treatments (4.81–5.74; Supplementary Table S1b).

Similarly to Berg and Smalla (2009), we also found significant differences in microbial structure between the vegetated and un-vegetated samples, but the change was only in the relative abundance of microbes at all studied levels (Schreiter et al. 2014). Plants can affect microbial biomass and diversity in several ways, but mostly through rhizodeposits (Raaijmakers et al. 2009, Neumann et al. 2014). As part of a related study, we found that *C. acuta* exudes mostly organic acids (unpublished data), which would increase soil microbial biomass and abundance (the priming effect; Jones et al. 2009, Kuzyakov 2010). Numerous bacteria, especially Acidobacteria, Actinobacteria, and alpha-Proteobacteria, were positively affected by the likely increased nutrient supply provided by the plants in our study, as also found by Mendes et al. (2013), Schreiter et al. (2014), and Kielak et al. (2016).

The known ability of *C. acuta* to exude oxygen from its roots may be a more important method by which *C. acuta* affects microbial abundance and structure in its rhizosphere (Visser et al. 2000, Colmer 2003). Radial oxygen leakage (ROL) occurs mostly through an increase in root porosity and the formation of adventitious roots in *C. acuta* (Colmer 2003). The leakage of oxygen would affect the soil redox state in the rhizosphere, which can greatly impact microbial community structure and functioning (Lamers et al. 2012, Tian et al. 2015), as noted by the lower relative abundances of typical anaerobic bacterial groups, such as Chloroflexi, Bacteriodetes, and Firmicutes, as well as methanogenic archaea, in the presence of plants.

### Treatment interactions

The soil, water level, and plant factors not only had significant direct effects on the composition of the soil microbial community, but also interacted to differentially impact soil microbial abundances at all of the analyzed levels, in agreement with our second hypothesis. Soil * water, soil * plant, and water * plant were the main two-factor interactions, while significant three-way interactions (soil * water * plant) were important in affecting the relative abundance of several of the Euryarchaeota, Actinobacteria, and delta-Proteobacteria families (Table 1). The soil * plant interaction is a common two way interaction in many studies since both soil factors and plants clearly affect microbial community structure and functioning (Berg and Smalla 2009, Schreiter et al. 2014). Significant water * plant interactions occurred through the coupling of saturated water conditions, with the increased possibility of the onset of anaerobic conditions, with the ability of *C. acuta* to oxygenate its rhizosphere (Visser et al. 2000, Colmer 2003). Examples of this interaction include the methanogenic archaea (Supplementary Fig. S5a) and iron-reducing bacteria (FRB; Supplementary Fig. S5d).

Even though there was no significant nutrient effect, our results show that nutrient additions work through plants to affect soil microbial abundance and diversity (significant fertilization * plant interaction). This interaction was especially important for particular Euryarchaeota, Acidobacteria, Actinobacteria, and alpha-, beta-, and delta-Proteobacteria families (Table 1). For most of these families, plant presence was a more important factor than nutrient addition, as evidenced by the significantly greater absolute abundances when plants were either present or absent (e.g. the Euryarchaeota), no matter the nutrient addition level. The exceptions were the Actinobacteria families C111 and Pseudonocardiaceae, in which the nutrient treatment was the differentiating factor either when plants were present (C111) or absent (Pseudonocardiaceae).

Both r- and K-strategists were important soil microbial members, noting that our system was likely quite dynamic (Mastný et al. 2021), although K-strategist families far outnumbered r-strategists . However, only two factor interactions (soil * water and water * plant) significantly affected these two strategic groups, with the number of K-strategist families being significantly greater in low-water peat and vegetated low-water conditions, respectively (Table 1). Contrary to hypothesis 3, we also found no indication of successional change, as occurs during DOC decomposition of peat (Mastný et al. 2021).

### Functional groups

The sequencing analyses showed that the C and Fe cycles were potentially the most important nutrient cycles in our experimental system at the family level, while the N cycle was of lesser importance. This finding is in agreement with other studies (Laanbroek 2010) showing close links between the C and Fe cycles in wetlands, with ferric iron reduction being a major C sink (Neubauer et al. 2005, Sutton-Grier and Megonigal 2011, Yarwood 2018), and possibly being an important component in SOM formation (Lalonde et al. 2012).

In our study, microbes possibly associated with the C cycle included methanogens (Euryarchaeota), methanotrophs, most notably members of the gamma-Proteobacteria Methylococcaceae and the alpha-Proteobacteria Methylocystaceae families (Knief 2019), and those capable of organic matter degradation (Haichar et al. 2007, Schellenberger et al. 2010, Kielak et al. 2016, Wieczorek et al. 2019) (Supplementary Fig. S5a–c). Likewise, microbes in our study possibly associated with Fe(III) reduction, most notably Firmicutes and Proteobacteria families, were more abundant in HW conditions but equally preferring vegetated or un-vegetated soils (Supplementary Fig. 5d). Iron oxidation and reduction are important cycles in wetlands (Mitsch and Gosselink 2000; Weiss et al. 2005, Yarwood 2018); therefore, it is not surprising that these may have a high abundance in our experimental system.

Diazotrophs (Acidobacteria, Actinobacteria, Firmicutes, and various Proteobacteria families) were the most abundant group of microbes in our study potentially associated with the N cycle, followed by the nitrifying archaea (Crenarchaeota; Supplementary Fig. S5e, f). Both groups, along with the OM-degrading bacteria, provide nutrients to support plant growth, thereby supporting hypothesis 4. The very low abundance of possible denitrifying bacteria may be connected to the great capacity of temperate wetland plants to take up large amounts of N (Yarwood 2018); however, this would need to be tested (Llado Fernandez et al. 2019).

The large variation seen in the abundances of the most common phyla shows the context-dependence of soil microbial abundance and how changing environmental conditions can significantly affect soil microbial community structure (Zhao et al. 2019). Interactions between the treatment factors emphasize that such changes to microbial community structure, and thus the functions that would be expected to be supported, are not straight-forward nor easily predictable. These findings have important implications for restoring or managing wet grasslands, as well as wetlands and other ecological systems in general.

## Conclusions

1. Soil type, water level, and plant presence or absence had the largest impacts on soil microbial community structure. Surprisingly, nutrient addition did not significantly directly impact the soil microbiome in our study.
2. The experimental factors affected soil microbial community structure both singly and interactively. The significantly reduced abundance of methanogens and methanotrophs in vegetated soils indicates the ability of *C. acuta* to oxidize its rhizosphere.
3. The lack of a change in r- and K-strategist presence and abundance (even under increased C inputs) did not support our third hypothesis that r-strategists would be favored in nutrient-richer and moist, but not saturated, conditions.
4. The abundance of possible plant growth-promoting bacteria (PGPB) and heterotrophic bacteria indicates the importance of bacteria that promote plant growth, thereby supporting hypothesis 4.

Changed precipitation patterns, as a result of climate change, are expected to affect ecosystem hydrology, structure, functioning, and the presence and abundance of numerous plant species (Maestra et al. 2012); however, we expect that *C. acuta* will remain a co-dominant species in Central European wet grasslands (Edwards and Čížková 2020). A drier and warmer future would result in nutrient-richer conditions, and changes to microbial community structure and total microbial biomass and/or abundances, with wet grasslands likely switching from areas acting as C sinks to C sources, while the opposite would be expected under wetter, more flooded conditions (Joyce et al. 2016).

## Supplementary Material

fiad070_Supplemental_Files
