# Supplementary material for: Multiple environmental factors, but not nutrient addition, directly affect wet grassland soil microbial community structure: a mesocosm study
Source: FEMS Microbiol Ecol. 2023 Jun 24;99(7):fiad070. doi: 10.1093/femsec/fiad070 (PMC10373907; doi:10.1093/femsec/fiad070)
Supplement: fiad070_Supplemental_Files [file fiad070_supplemental_files.zip › Supp_data Figure Legends.docx]

**Supplementary Figure Legends**

Figure S1. Relative abundance of the Archaea phyla (average from four sampling dates in March, May, July and September 2013; n = 8) found in either peat soil (a) or mineral soil (b) under different treatment conditions in a mesocosm experiment. Treatments: water level: LW = Low (15cm below soil surface), HW = high (saturated, water level maintained at the soil surface); fertilization: UF = unfertilized (0 kg NPK ha^-1^ yr^-1^), F = fertilized (300 kg NPK ha^-1^ yr^-1^); plants: UV = un-vegetated, V = vegetated.

Figure S2. PCA graphs of selected Archaea classes; see Methods for selection criteria. Axis 1 (explained data variance = 35.56 %) was related to soil type while water level was more correlated with axis 2 (explained variance = 21.99%). A: Samples with treatment combination centroids; B: Treatment centroids with vectors showing the strength of relations between the Archaea classes and the axes. Factor abbreviations: Soil*Water*Plants (SWP): Soil type (S: P = peat; M= mineral); Water level (W: LW = low, 15 cm below soil surface; HW = high, saturated, water level at soil surface); Plants (P: Unveg = un-vegetated; Veg = vegetated). Taxa abbreviations: MethBact = Methanobacteria; MethMicr = Methanomicrobia; Parva = Parvaarchaea; Thaum = Thaumarchaeota; Thermo = Thermoplasmata.

Figure S3. First two axes of a PCA from OTU data of the selected bacteria phyla including the alpha-, beta-, delta- and gamma-Proteobacteria classes; see Methods for selection criteria. Axis 1 (explained data variance = 55.11 %) was related to water level while soil type was more correlated with axis 2 (explained variance = 19.35%). A: Samples with treatment combination centroids; B: Treatment centroids with vectors showing the strength of relations between the bacterial phyla plus Proteobacteria classes and the axes. Factor abbreviations: Soil*Water*Plants (SWP): Soil type (S: P = peat; M= mineral); Water level (W: LW = low, 15 cm below soil surface; HW = high, saturated, water level at soil surface); Plants (P: Unveg = un-vegetated; Veg = vegetated). Phyla abbreviations: Acido = Acidobacteria; Actino = Actinobacteria; Bacter = Bacteroidetes; Cyano = Cyanobacteria; Firmi = Firmicutes; Gemmati = Gemmatimonadetes; Plancto = Planctomycetes; Verru = Verrucomicrobia; AlphaPro = Alpha Proteobacteria; BetaPro = Beta Proteobacteria; DeltaPro = Delta Proteobacteria; GammaPro = Gamma Proteobacteria.

Figure S4. First two axes of a PCA from OTU data of selected bacterial families; see Methods for selection criteria. Axis 1 (explained data variance = 29.72 %) was related to soil type while water level was more correlated with axis 2 (explained variance = 28.72%). Symbols represent the samples used in the PCA. Factor abbreviations: Soil*Water*Plants (SWP): Soil type (S: P = peat; M= mineral); Water level (W: LW = low, 15 cm below soil surface; HW = high, saturated, water level at soil surface); Plants (P: Unveg = un-vegetated; Veg = vegetated)

Figure S5. Mean absolute abundances (measured as OTUs; OTU.g^-1^ dw soil, black bars) and proportion of total microbial abundance (striped bars) from four sampling times (March, May, July and September 2013) for selected functional groups; see Methods for selection criteria. Functional groups: Carbon cycle: A) Methanogens; B) Methanotrophs; C) Organic matter (OM) degraders; Iron cycle: D) Iron reducing bacteria (FRB); Nitrogen cycle: E) Diazotrophs; F) Nitrifiers. Treatments: soil type = peat, mineral; water level: LW (low = 15cm below soil surface), HW (high = saturated, water level maintained at the soil surface); nutrients: UF (unfertilized = 0 kg NPK ha^-1^ yr^-1^), F (fertilized = 300 kg NPK ha^-1^ yr^-1^); plants: UV = un-vegetated; V = vegetated. Note range differences on the Y-axes.
