# Supplementary material for: Multiple environmental factors, but not nutrient addition, directly affect wet grassland soil microbial community structure: a mesocosm study
Source: FEMS Microbiol Ecol. 2023 Jun 24;99(7):fiad070. doi: 10.1093/femsec/fiad070 (PMC10373907; doi:10.1093/femsec/fiad070)
Supplement: fiad070_Supplemental_Files [file fiad070_supplemental_files.zip › Supp_data Table_S1.docx]

**Table S1. A) Initial conditions of the developed peat and mineral soils. B) Effect of different factors (soil type, water level, fertilization, plant presence) on mean soil pH, soluble organic carbon (SOC, μg.g^-1^ dw soil) and total soluble nitrogen (TSN, μg.g^-1^ dw soil) levels (averaged over four sampling times in March, May, July and September 2013) and plant net aboveground and belowground primary production (NAPP and NBPP, respectively) after five years of experimental treatments (2013 only). Treatment acronyms: Soil type (Peat; Min=mineral); water level (LW=low water; HW=high water); fertilization (UF=unfertilized (0 kg NPK ha^-1^ yr^-1^); F = fertilized (300 kg NPK ha^-1^ yr^-1^); plants (UV= un-vegetated; V=vegetated).**

**A)**

| **Factor** | **Peat** | **Mineral** |
| --- | --- | --- |
| **C_tot_, mg g^-1^** | **139.4 ± 0.4** | **12.7 ± 0.2** |
| **N_tot_, mg g^-1^** | **5.39 ± 0.05** | **0.58 ± 0.04** |
| **P_tot_, mg g^-1^** | **0.41 ±0.02** | **0.39 ± 0.03** |
| **C:N** | **25.8** | **22.1** |
| **C:P** | **344** | **32** |
| **pH – H_2_O** | **4.46 ± 0.04** | **6.08 ± 0.01** |
| **Bulk Density, g cm^-3^** | **0.49** | **1.49** |

**B)**

| **Treatments** | | | | **pH** | **SOC** | **TSN** | **NAPP/NBPP** |
| --- | --- | --- | --- | --- | --- | --- | --- |
| **Peat** | **LW** | **UF** | **UV** | 5.60±0.41 | 64.31±18.05 | 14.05±8.63 | -------- |
|  |  |  | **V** | 5.31±0.22 | 101.88±18.18 | 13.51±8.65 | 1210.25/970.52 |
|  |  | **F** | **UV** | 5.17±0.30 | 79.28±23.00 | 72.80±74.71 | -------- |
|  |  |  | **V** | 4.81±0.33 | 168.87±38.90 | 17.30±10.69 | 1251.25/1379.13 |
|  | **HW** | **UF** | **UV** | 5.74±0.44 | 46.09±22.79 | 35.82±12.83 | -------- |
|  |  |  | **V** | 5.34±0.16 | 82.95±21.06 | 13.41±12.08 | 533.50/1286.23 |
|  |  | **F** | **UV** | 5.72±0.20 | 44.81±14.27 | 55.75±29.80 | -------- |
|  |  |  | **V** | 5.09±0.21 | 117.46±23.02 | 13.05±8.02 | 938.75/1643.52 |
| **Min** | **LW** | **UF** | **UV** | 6.08±0.28 | 21.17±9.73 | 10.97±5.14 | -------- |
|  |  |  | **V** | 6.12±0.19 | 25.40±8.63 | 5.90±4.42 | 507.50/537.23 |
|  |  | **F** | **UV** | 6.11±0.28 | 23.52±7.14 | 19.51±10.90 | -------- |
|  |  |  | **V** | 6.11±0.33 | 33.37±14.64 | 9.55±6.69 | 1061.75/1513.60 |
|  | **HW** | **UF** | **UV** | 6.15±0.16 | 19.97±10.08 | 22.01±7.49 | -------- |
|  |  |  | **V** | 5.90±0.32 | 37.41±15.56 | 7.93±4.64 | 439.75/552.17 |
|  |  | **F** | **UV** | 6.04±0.29 | 23.51±12.41 | 29.41±12.18 | -------- |
|  |  |  | **V** | 5.84±0.30 | 30.15±18.11 | 8.04±4.23 | 993.00/995.21 |
