# Supplementary material for: Multiple environmental factors, but not nutrient addition, directly affect wet grassland soil microbial community structure: a mesocosm study
Source: FEMS Microbiol Ecol. 2023 Jun 24;99(7):fiad070. doi: 10.1093/femsec/fiad070 (PMC10373907; doi:10.1093/femsec/fiad070)
Supplement: fiad070_Supplemental_Files [file fiad070_supplemental_files.zip › Supp_data Table_S2.docx]

**Table S2. List of all selected Archaea and bacterial phyla, classes and families used in the analyses and their r/K strategy using the PICRUSt pipeline (Langille et al. 2013). * classified into a functional group.**

| **Kingdom** | **Phylum** | **Class** | **Family** | **r/k** |
| --- | --- | --- | --- | --- |
| Archaea | Crenarchaeota | Thaumarchaeota | Sagma-X * | k |
|  |  |  | Nitrososphaeraceae * | k |
|  |  |  |  |  |
|  | Euryarchaeota | Methanobacteria | Methanobacteriaceae * | k |
|  |  | Methanomicrobia | Methanocellaceae * | k |
|  |  |  | Methanoregulaceae * | k |
|  |  |  | Methanosaetaceae * | k |
|  |  |  | Methanosarcinaceae * | k |
|  |  | Thermoplasmata | Methanomassiliicoccaceae * | k |
|  |  |  |  |  |
|  |  |  |  |  |
| Bacteria | Acidobacteria | Acidobacteria-6 | RB40 | k |
|  |  | Acidobacteriia | Acidobacteriaceae | k |
|  |  |  | Koribacteraceae | k |
|  |  | Holophagae | Holophagaceae * | k |
|  |  | Solibacteres | AKIW659 | k |
|  |  |  | Solibacteraceae | k |
|  |  |  |  |  |
|  | Actinobacteria | Acidimicrobiia | C111 | k |
|  |  |  | EB1017 | k |
|  |  | Actinobacteria | Cellulomonadaceae * | k |
|  |  |  | Frankiaceae * | k |
|  |  |  | Intrasporangiaceae * | k |
|  |  |  | Kineosporiaceae * | r |
|  |  |  | Microbacteriaceae * | k |
|  |  |  | Micrococcaceae * | r |
|  |  |  | Micromonosporaceae * | k |
|  |  |  | Mycobacteriaceae * | k |
|  |  |  | Nakamurellaceae | k |
|  |  |  | Nocardiaceae | r |
|  |  |  | Pseudonocardiaceae * | r |
|  |  |  | Streptomycetaceae * | r |
|  |  |  | Thermomonosporaceae | r |
|  |  | Thermoleophilia | Gaiellaceae | k |
|  |  |  | Conexibacteraceae | k |
|  |  |  | Patulibacteraceae | k |
|  |  |  | Solirubrobacteraceae | k |
|  |  |  |  |  |
|  | Bacteroidetes | Bacteroidia | GZKB119 | r |
|  |  |  | Porphyromonadaceae | r |
|  |  |  | Rikenellaceae | k |
|  |  |  | SB-1 | r |
|  |  | Sphingobacteriia | Sphingobacteriaceae * | r |
|  |  | Saprospirae | Chitinophagaceae * | k |
|  |  |  | Saprospiraceae | k |
|  |  |  |  |  |
|  | Chlorobi | Ignavibacteriales | Ignavibacteriaceae * | k |
|  |  |  | Melioribacteraceae * | k |
|  |  |  |  |  |
|  | Chloroflexi | Anaerolineae | Anaerolinaceae * | k |
|  |  |  | Caldilineaceae | k |
|  |  |  | A4b | k |
|  |  |  | SHA-31 | k |
|  |  |  | oc28 | k |
|  |  | Ktedonobacteria | Ktedonobacteraceae | r |
|  |  |  | Thermogemmatisporaceae | r |
|  |  |  |  |  |
|  | Cyanobacteria | Chloroplast | Chlamydomonadaceae |  |
|  |  |  | Trebouxiophyceae |  |
|  |  | Nostocophycideae | Nostocaceae * | r |
|  |  |  |  |  |
|  | Firmicutes | Bacilli | Alicyclobacillaceae * | r |
|  |  |  | Bacillaceae * | r |
|  |  |  | Paenibacillaceae * | r |
|  |  |  | Planococcaceae | r |
|  |  | Clostridia | Christensenellaceae | k |
|  |  |  | Clostridiaceae * | r |
|  |  |  | Gracilibacteraceae | r |
|  |  |  | Lachnospiraceae | r |
|  |  |  | Peptococcaceae | r |
|  |  |  | Ruminococcaceae | r |
|  |  |  | Veillonellaceae * | r |
|  |  |  |  |  |
|  | Gemmatimonadetes | Gemmatimonadetes | Ellin5301 | k |
|  |  |  | Gemmatimonadaceae | k |
|  |  |  |  |  |
|  | Planctomycetes | Planctomycetia | Gemmataceae | k |
|  |  |  | Isosphaeraceae | r |
|  |  |  | Pirellulaceae * | k |
|  |  |  | Planctomycetaceae * | k |
|  |  |  |  |  |
|  | Proteobacteria | Alphaproteobacteria | Caulobacteraceae | k |
|  |  |  | Beijerinckiaceae * | k |
|  |  |  | Bradyrhizobiaceae * | k |
|  |  |  | Hyphomicrobiaceae * | k |
|  |  |  | Methylocystaceae * | k |
|  |  |  | Rhodobacteraceae * | k |
|  |  |  | Acetobacteraceae * | r |
|  |  |  | Rhodospirillaceae * | r |
|  |  |  | Sphingomonadaceae * | k |
|  |  |  |  |  |
|  |  | Betaproteobacteria | EB1003 | k |
|  |  |  | Alcaligenaceae * | k |
|  |  |  | Burkholderiaceae * | r |
|  |  |  | Comamonadaceae * | k |
|  |  |  | Oxalobacteraceae * | r |
|  |  |  | Gallionellaceae * | k |
|  |  |  | Methylophilaceae | k |
|  |  |  | Rhodocyclaceae * | k |
|  |  |  |  |  |
|  |  | Deltaproteobacteria | Bdellovibrionaceae | k |
|  |  |  | Desulfobulbaceae * | r |
|  |  |  | Geobacteraceae * | k |
|  |  |  | Haliangiaceae * | k |
|  |  |  | Myxococcaceae * | r |
|  |  |  | Polyangiaceae | r |
|  |  |  | Syntrophaceae * | k |
|  |  |  | Syntrophobacteraceae * | k |
|  |  |  |  |  |
|  |  | Gammaproteobacteria | Coxiellaceae | k |
|  |  |  | Legionellaceae | k |
|  |  |  | Crenotrichaceae | k |
|  |  |  | Methylococcaceae * | k |
|  |  |  | Pseudomonadaceae | r |
|  |  |  | Piscirickettsiaceae | r |
|  |  |  | Sinobacteraceae | k |
|  |  |  | Xanthomonadaceae * | k |
|  |  |  |  |  |
|  | Verrucomicrobia | Opitutae | Opitutaceae | k |
|  |  | Pedosphaerae | Ellin515 | k |
|  |  |  | Pedosphaeraceae | k |
|  |  |  | auto67_4W | k |
|  |  | Spartobacteria | Chthoniobacteraceae | k |
