# Supplementary material for: Multiple environmental factors, but not nutrient addition, directly affect wet grassland soil microbial community structure: a mesocosm study
Source: FEMS Microbiol Ecol. 2023 Jun 24;99(7):fiad070. doi: 10.1093/femsec/fiad070 (PMC10373907; doi:10.1093/femsec/fiad070)
Supplement: fiad070_Supplemental_Files [file fiad070_supplemental_files.zip › Supp_data Table_S3.docx]

**Table S3. List of archaea and bacteria families that comprised the different microbial functional groups.**

| **Functional Groups** | **Phylum** | **Class** | **Family** |
| --- | --- | --- | --- |
| **C cycle** |  |  |  |
| Methanogens | Euryarchaeota | Methanobacteria | Methanobacteriaceae |
|  |  | Methanomicrobia | Methanocellaceae |
|  |  |  | Methanoregulaceae |
|  |  |  | Methanosaetaceae |
|  |  |  | Methanosarcinaceae |
|  |  | Thermoplasmata | Methanomassiliicoccaceae |
| Methanotrophs | Proteobacteria | Alphaproteobacteria | Methylocystaceae |
|  |  | Gammaproteobacteria | Methylococcaceae |
| OM Degraders | Acidobacteria | Acidobacteriia | Acidobacteriaceae |
|  |  |  | Koribacteraceae |
|  |  | Solibacteres | Solibacteraceae |
|  | Actinobacteria | Actinobacteria | Cellulomonadaceae |
|  |  |  | Intrasporangiaceae |
|  |  |  | Kineosporiaceae |
|  |  |  | Microbacteriaceae |
|  |  |  | Micrococcaceae |
|  |  |  | Micromonosporaceae |
|  |  |  | Mycobacteriaceae |
|  |  |  | Nocardioidiaceae |
|  |  |  | Pseudonocardiaceae |
|  |  |  | Streptomycetaceae |
|  | Bacteroidetes | Sphingobacteriia | Sphingobacteriaceae |
|  |  | Saprospirae | Chitinophagaceae |
|  | Firmicutes | Bacilli | Bacillaceae |
|  |  | Clostridia | Clostridiaceae |
|  |  |  | Ruminococcaceae |
|  |  |  | Veillonellaceae |
|  | Planctomycetes | Planctomycetia | Pirellulaceae |
|  |  |  | Planctomycetaceae |
|  | Proteobacteria | Alphaproteobacteria | Hyphomicrobiaceae |
|  |  |  | Sphingomoandaceae |
|  |  | Betaproteobacteria | Oxalobacteraceae |
|  |  | Deltaproteobacteria | Haliangiaceae |
|  |  |  | Myxococcaceae |
|  |  | Gammaproteobacteria | Pseudomonadaceae |
|  |  |  | Xanthomonadaceae |
|  | Verrucomicrobia | Opitutae | Opitutaceae |
| **N cycle** |  |  |  |
| N Fixers | Actinobacteria | Actinobacteria | Frankiaceae |
|  |  |  | Microbacteriaceae |
|  | Cyanobacteria | Nostocophycideae | Nostocaceae |
|  | Firmicutes | Bacilli | Paenibacillaceae |
|  | Proteobacteria | Alphaproteobacteria | Beijerinckiaceae |
|  |  |  | Bradyrhizobiaceae |
|  |  |  | Acetobacteraceae |
|  |  |  | Rhodospirillaceae |
|  |  |  | Sphingomonadaceae |
|  |  | Betaproteobacteria | Burkholderiaceae |
|  |  |  | Rhodocyclaceae |
| Nitrifiers | Crenarchaeota | Thaumarchaeota | SAGMA-X |
|  |  |  | Nitrososphaeraceae |
|  | Proteobacteria | Alphaproteobacteria | Bradyrhizobiaceae |
| **Iron Cycle** |  |  |  |
| FRB | Acidobacteria | Holophagae | Holophagaceae |
|  | Chlorobi | Ignavibacteria | Ignavibacteriaceae |
|  |  |  | Melioribacteraceae |
|  | Chlroflexi | Anaerolineae | Anaerolinaceae |
|  | Firmicutes | Bacilli | Alicyclobacillaceae |
|  |  | Clostridia | Clostridiaceae |
|  |  |  | Peptococcaceae |
|  | Proteobacteria | Alphaproteobacteria | Acetobacteraceae |
|  |  |  | Rhodospirillaceae |
|  |  | Betaproteobacteria | Burkholderiaceae |
|  |  |  | Comamonadaceae |
|  |  | Deltaproteobacteria | Desulfobulbaceae |
|  |  |  | Geobacteraceae |
|  |  |  | Myxococcaceae |
