# Supplementary figures and images for: Multiple environmental factors, but not nutrient addition, directly affect wet grassland soil microbial community structure: a mesocosm study
Source: FEMS Microbiol Ecol. 2023 Jun 24;99(7):fiad070. doi: 10.1093/femsec/fiad070 (PMC10373907; doi:10.1093/femsec/fiad070)

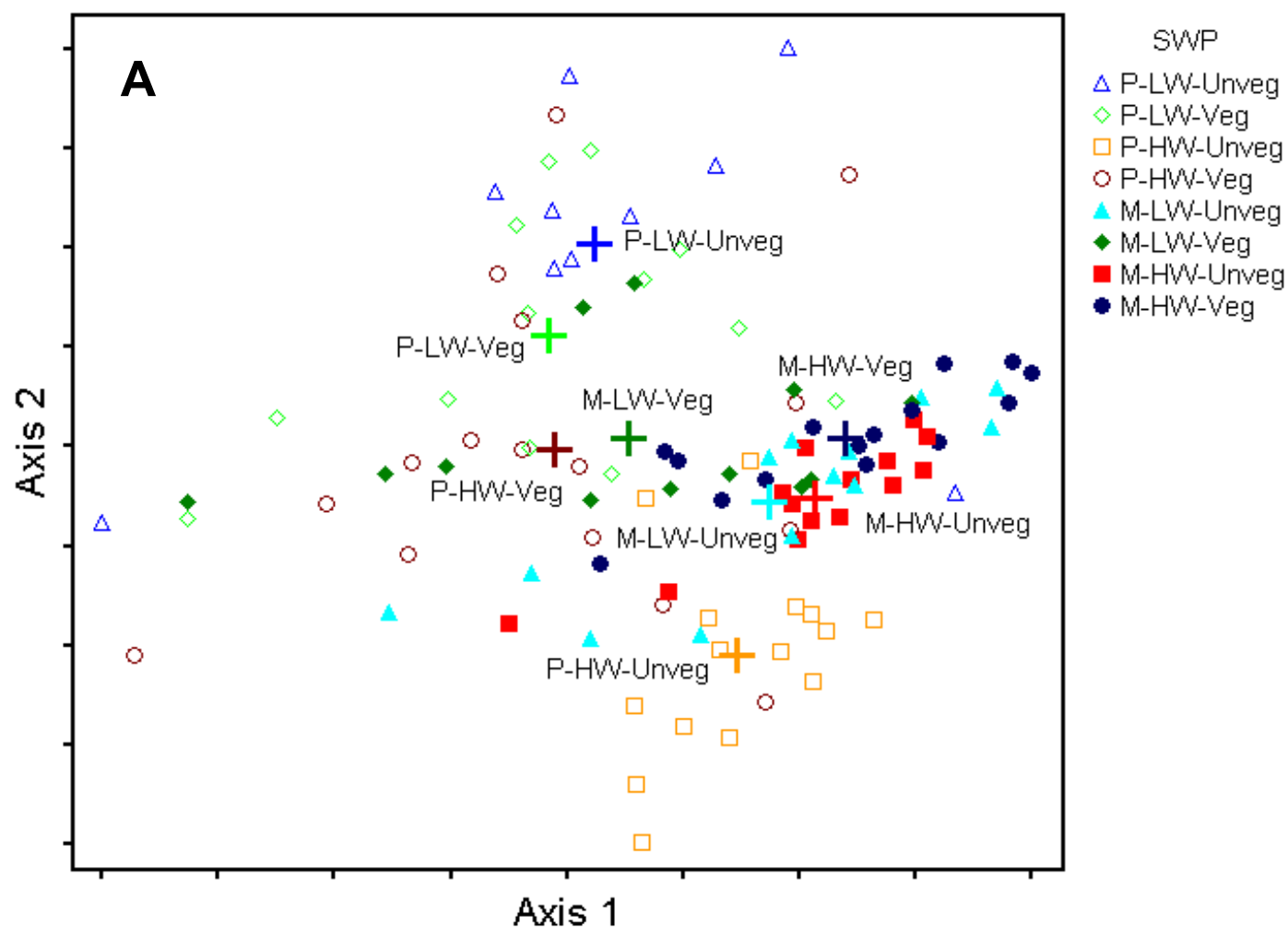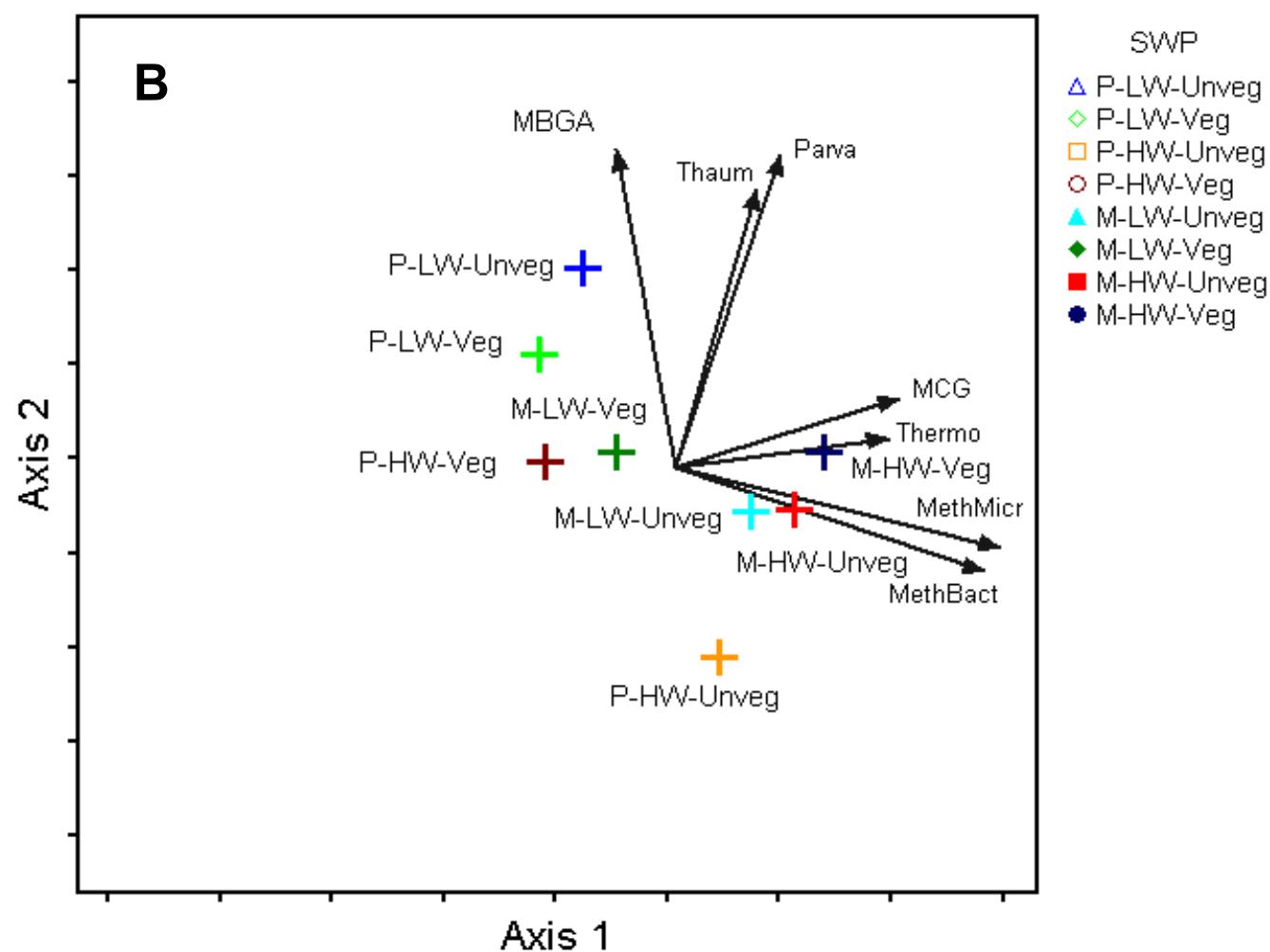

Supplement: fiad070_Supplemental_Files [file fiad070_supplemental_files.zip › Supp_data Figure 2.pdf]

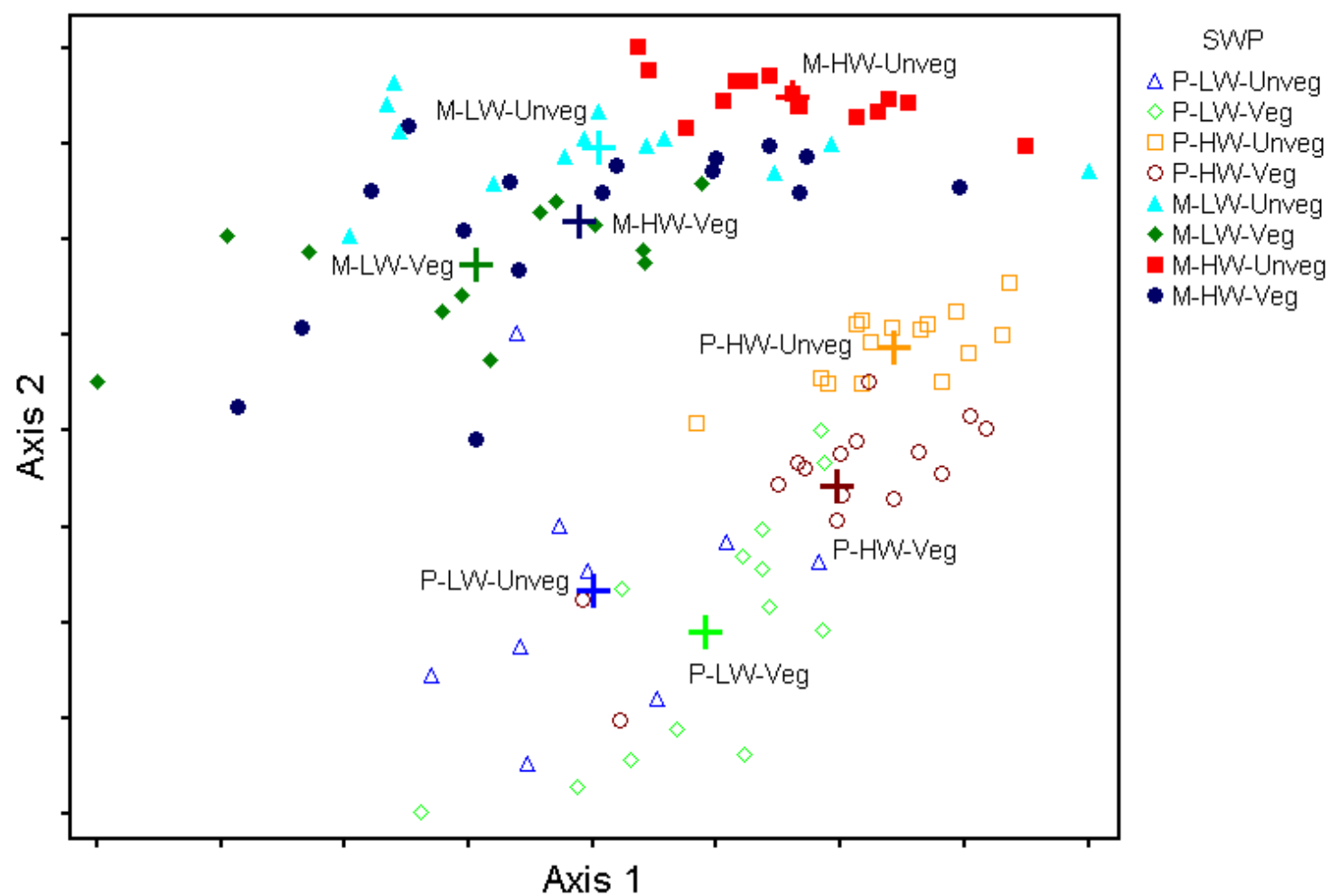

Supplement: fiad070_Supplemental_Files [file fiad070_supplemental_files.zip › Supp_data Figure 4.pdf]

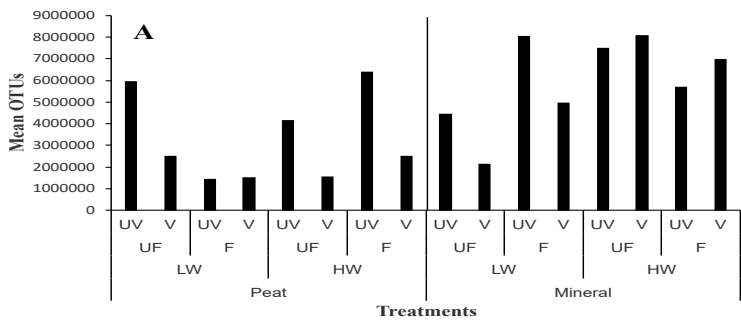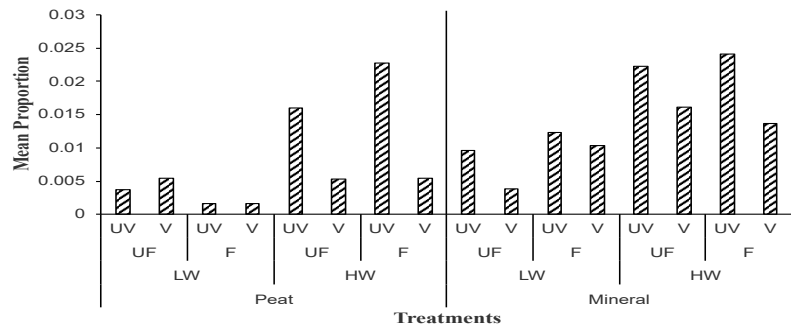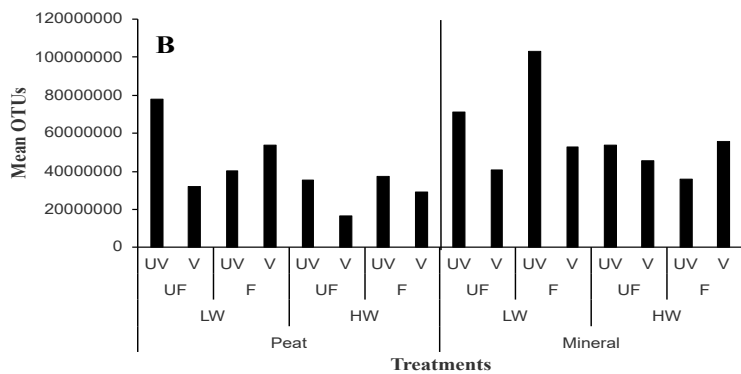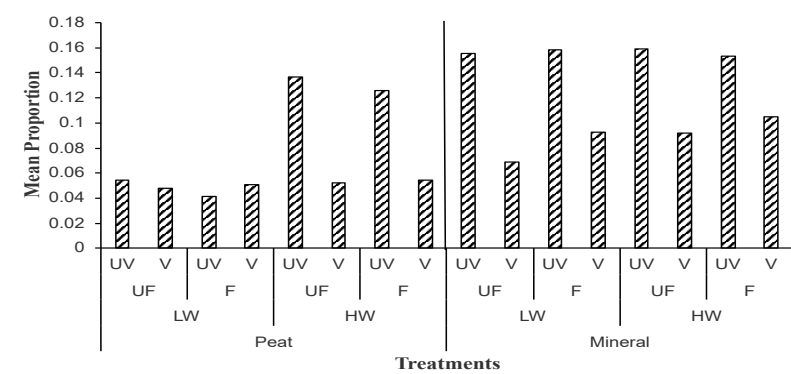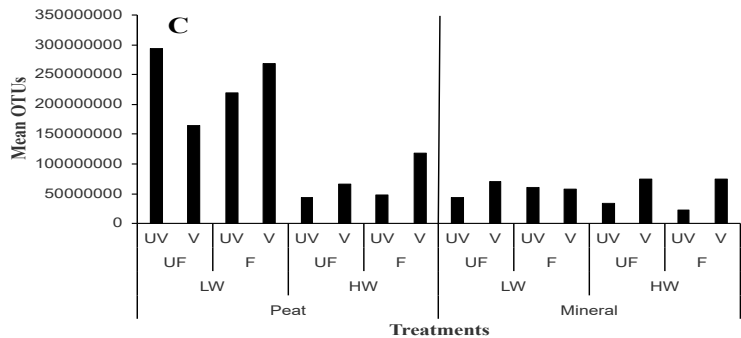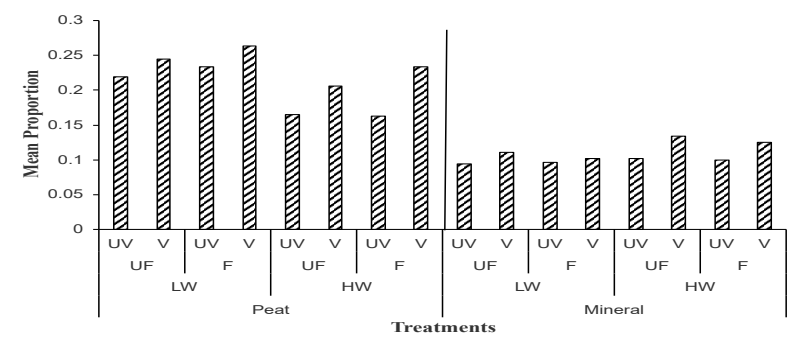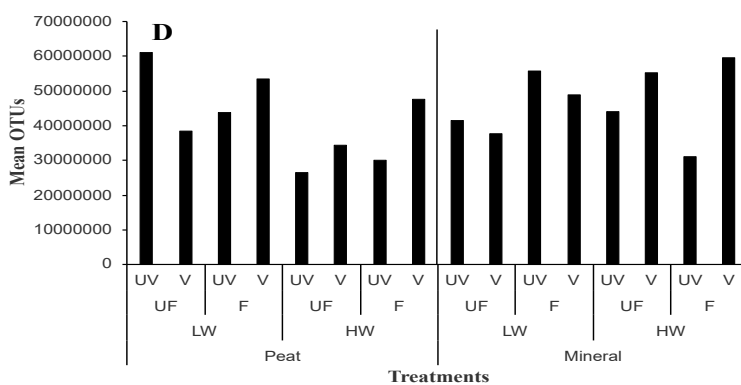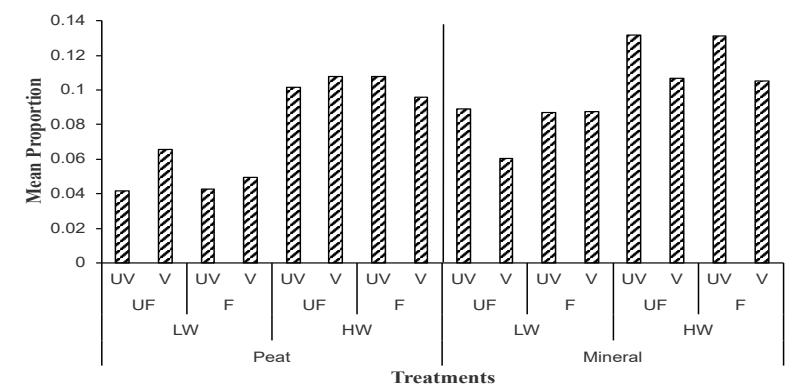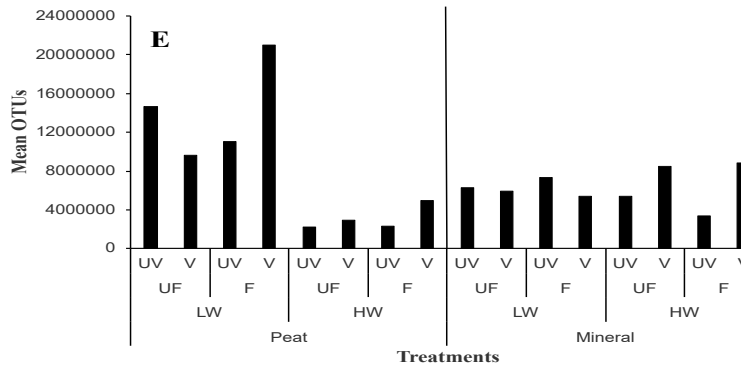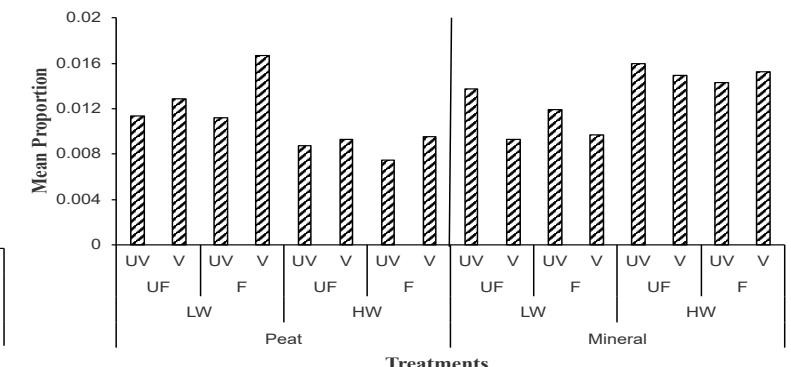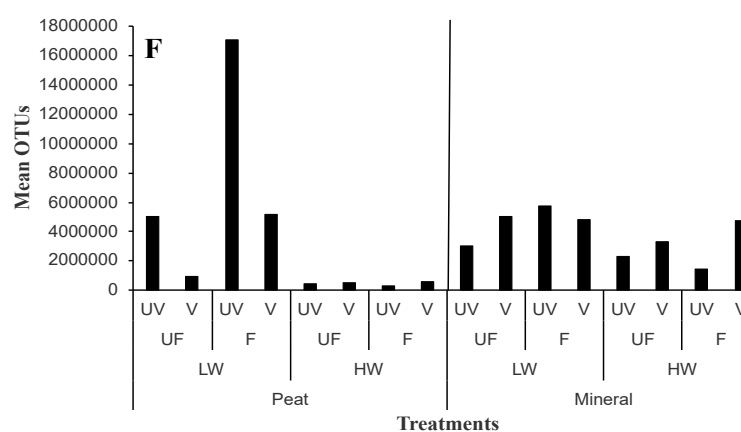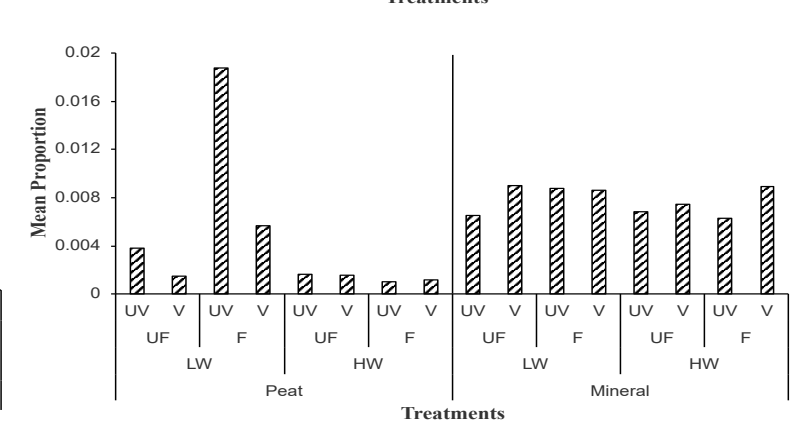

Supplement: fiad070_Supplemental_Files [file fiad070_supplemental_files.zip › Supp_data Figure 5.pdf]

**A**

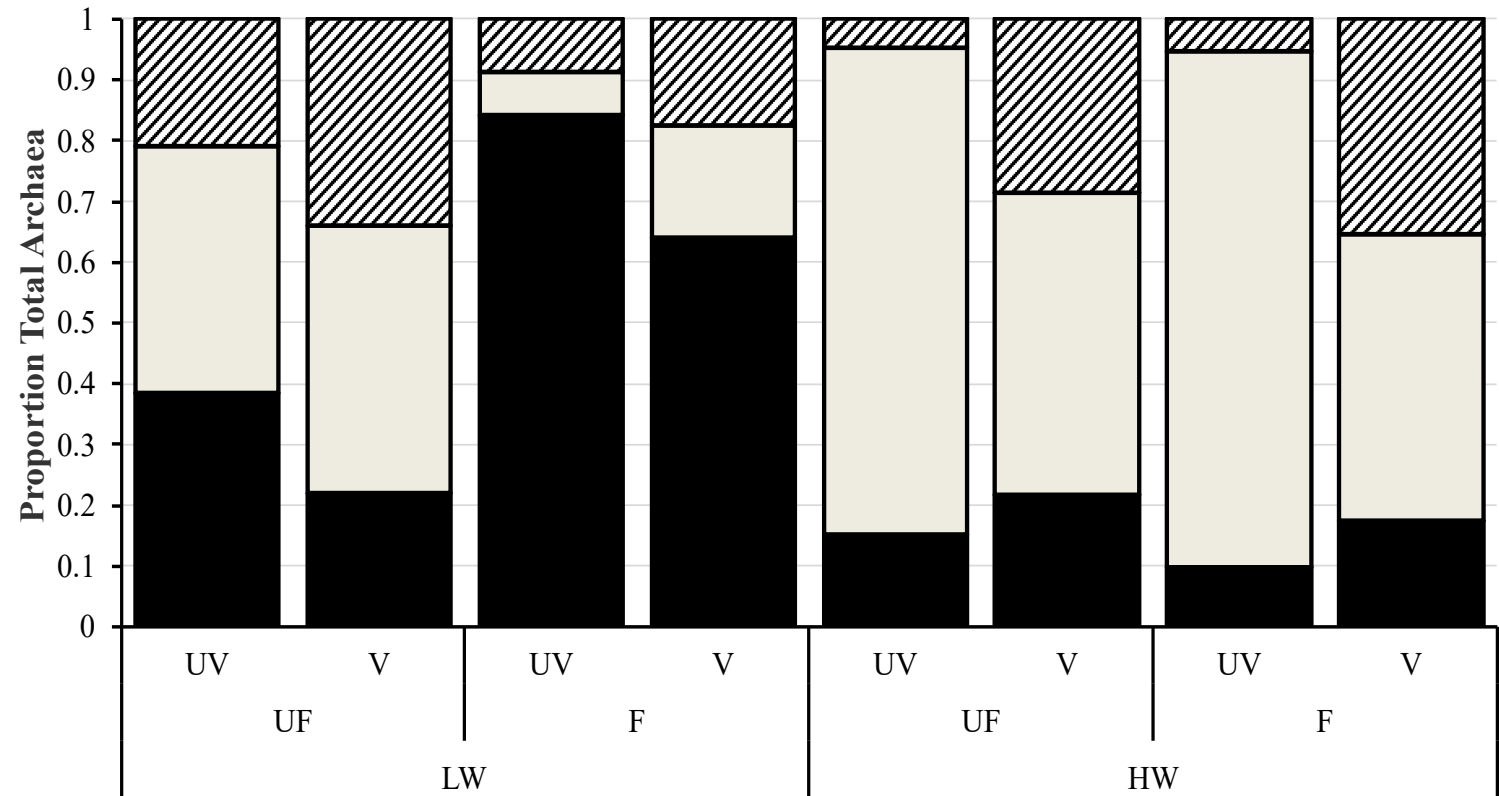

**Peat soil**

**B**

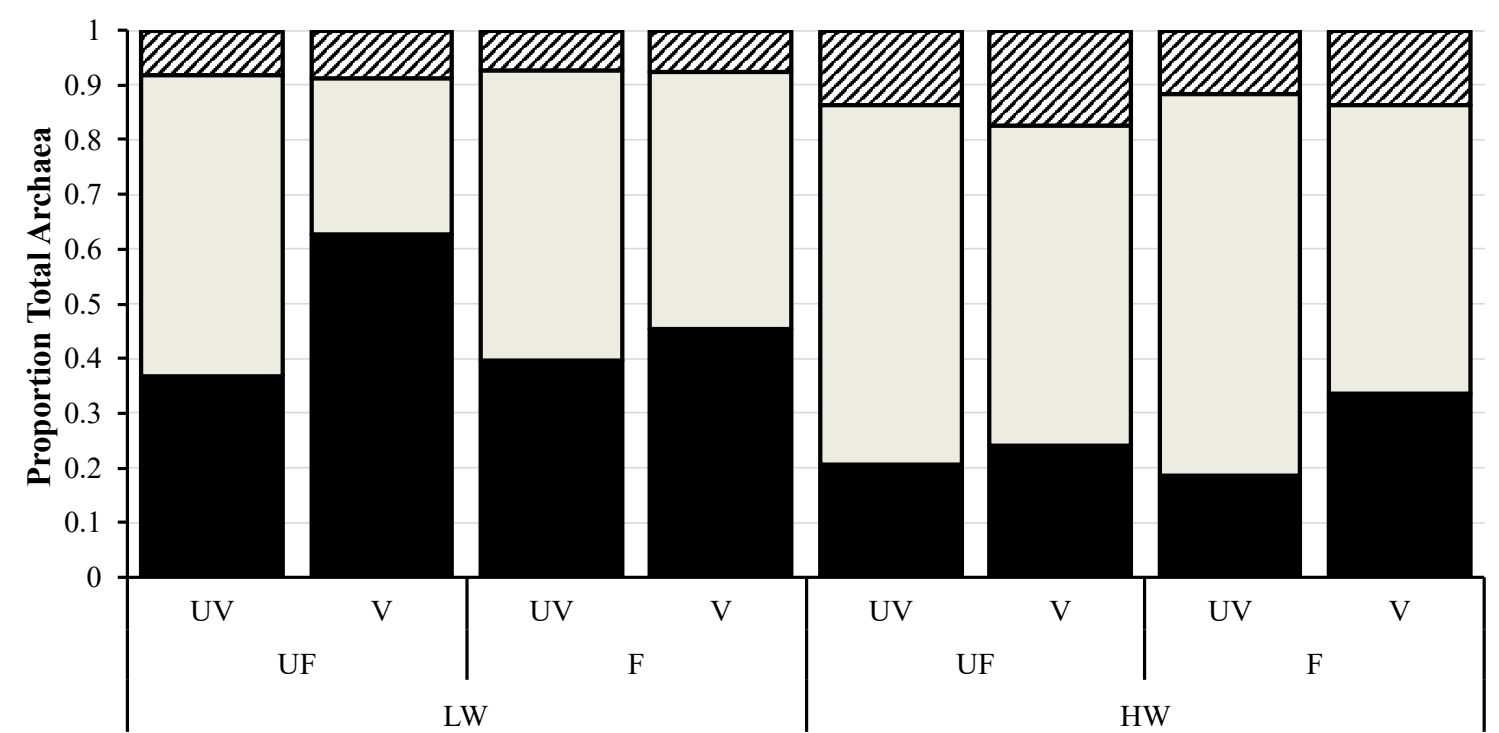

**Mineral soil**

■ Crenarchaeota    □ Euryarchaeota    ▨ Parvarchaeota

Supplement: fiad070_Supplemental_Files [file fiad070_supplemental_files.zip › Supp_data Figure 1.pdf]

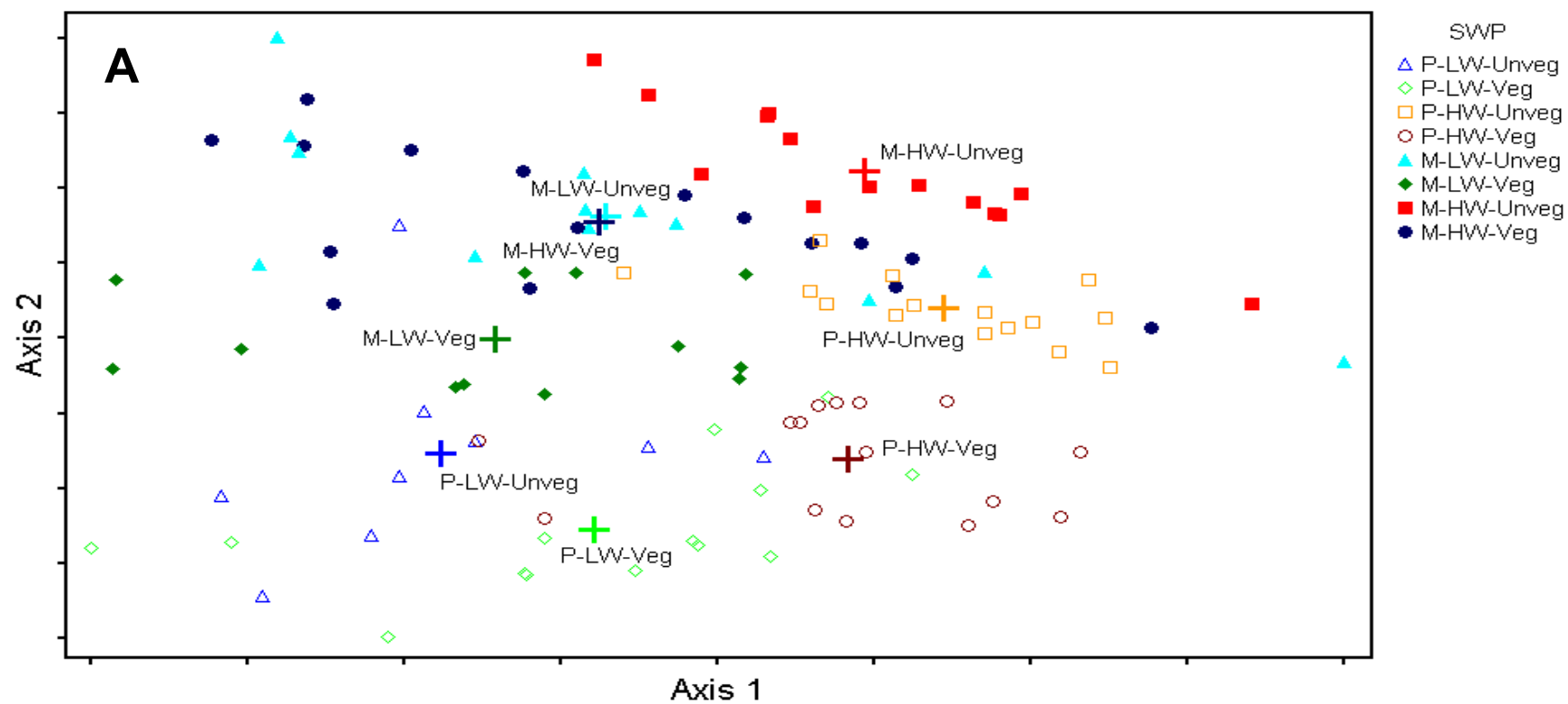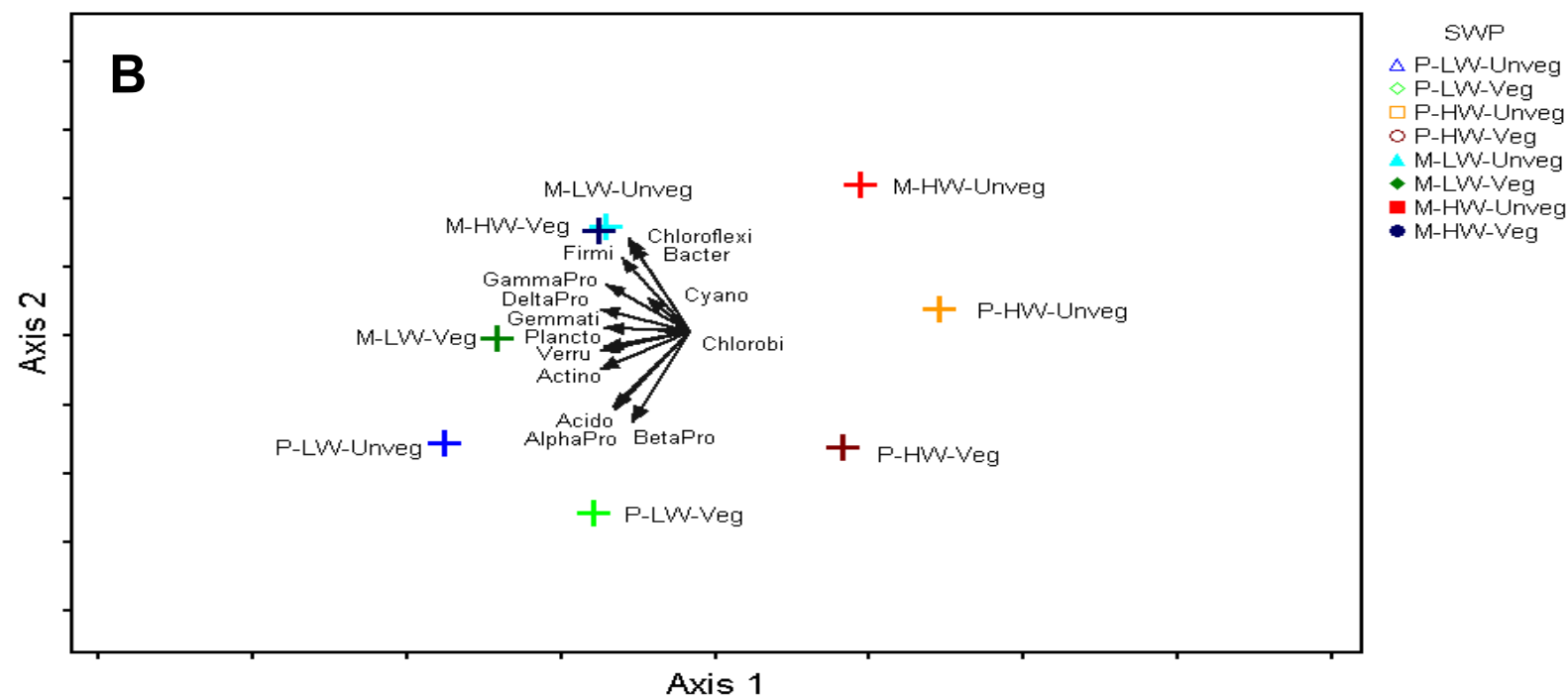

Supplement: fiad070_Supplemental_Files [file fiad070_supplemental_files.zip › Supp_data Figure 3.pdf]
